# Adsorption of bentazone in the profiles of mineral soils with low organic matter content

**DOI:** 10.1371/journal.pone.0242980

**Published:** 2020-12-02

**Authors:** Tadeusz Paszko, Joanna Matysiak, Daniel Kamiński, Sylwia Pasieczna-Patkowska, Miłosz Huber, Beata Król

**Affiliations:** 1 Department of Chemistry, University of Life Sciences, Lublin, Poland; 2 Department of General and Coordination Chemistry and Crystallography, Maria Curie-Skłodowska University, Lublin, Poland; 3 Department of Chemical Technology, Maria Curie-Skłodowska University, Lublin, Poland; 4 Department of Geology, Soil Science and Geoinformation, Maria Curie-Skłodowska University, Lublin, Poland; 5 Department of Industrial and Medicinal Plants, University of Life Sciences, Lublin, Poland; Qatar University, QATAR

## Abstract

The current laboratory adsorption study aimed at determination of the values of adsorption distribution coefficient (*K*_*d*_) of bentazone in the profiles of Arenosols, Luvisols, and Cambisols, which are the most common arable mineral soils in Poland. The study attempted to identify the soil components that bind bentazone and the principal adsorption mechanisms of this compound as well as create a model capable of predicting its adsorption in soils. The *K*_*d*_ values determined in batch experiments after 24 h of shaking were very low, and ranged from 0.05 to 0.30 mL/g for the Ap horizon and 0 to 0.07 mL/g for subsoils. The results indicated that the anionic form of bentazone was adsorbed on organic matter, while in acidic soils the neutral form of bentazone was adsorbed on organic matter and sand. The detailed analyses of mineralogical composition revealed that the principal mineral that was responsible for the adsorption of bentazone was quartz, which content was strongly positively correlated with the sand fraction. In soils with pH < 5 and an organic carbon content of < 0.35%, quartz exhibited much greater affinity for the neutral bentazone form than organic matter. Fourier transform infrared photoacoustic spectroscopy analyses supported by computational methods have shown the most probable mechanisms behind the adsorption of bentazone on quartz. The created model, assuming the adsorption of bentazone on organic matter and on sand and using the spectrophotometrically determined dissociation constant of bentazone, very well explained the *K*_*d*_ variance in the 81 examined soils, while correctly predicting the adsorption based on soil properties described in the published data.

## Introduction

Bentazone (3-isopropyl-1*H*-2,1,3-benzothiadiazin-4(3*H*)-one 2,2-dioxide) is a post-emergence contact herbicide widely used all over the world. In the European Union (EU) it is used for selective management of broadleaf weeds and sedges in various plants such as corn, potato, rice, alfalfa, sorghum, linseed, peanuts, beans, peas, clover, chives, garlic, and ornamentals [1,2].

The compound is known to be highly soluble in water (7.1 g/L at 20°C), but weakly adsorbed in soils [1]. For example, in the EU dossier (25 soils) from 2015 [1], the Freundlich adsorption coefficient was shown to be in the range of 0.02–3.06 μg^1–1/n^ (mL)^1/n^ g^–1^ and the Freundlich exponent *1/n* in the range of 0.7–1.03. The adsorption of bentazone increases with an increase in the content of organic matter in soil [3–5]. Moreover, the level of adsorption of this weak organic acid is inversely proportional to the soil pH [5,6]. Organic matter present in soils is the predominant adsorbent of bentazone. Bonfleur et al. [7] showed that its adsorption is related to the ratio of alkyl-C/O-alkyl-C groups as well as the hydrophobicity of organic matter, determined by the ^13^C Nuclear Magnetic Resonance technique. On the other hand, the pattern of pH-dependent adsorption of bentazone on organic matter is consistent with the pattern of its logD values (logarithm of the octanol-water partition coefficient at specific pH) [4]. However, organic matter may not be the only soil component participating in adsorption. A study by Clausen et al. [8] indicated that at a low pH bentazone is adsorbed on quartz, while at a near-neutral pH it is adsorbed on α-alumina and kaolinite. The pH-dependent adsorption of bentazone on mesoporous silica (strong in the pH range of 2–4 and much weaker in the range of 5–7), as well as the weak adsorption of this compound on montmorillonite, were described by Bruzzoniti et al. [9]. Adsorption of bentazone on silica at pH of 6.5–7.5 was observed also by Spaltro et al. [10]. Furthermore, the adsorption of bentazone on synthesized iron oxides at a pH of 3.4–4.6 was stated by Clausen and Fabricius [11].

Despite its rather fast degradation (e.g., half-life of 8–35 days in 12 soils as indicated in the EU dossier [1]), bentazone is considered to exhibit medium-to-high mobility in soils. The pan-European surveys conducted by Loos et al. [12] and [13] revealed that bentazone was detected in 32% of groundwater samples and in 69% of river water samples, in some cases at concentrations exceeding the allowable limit put forth by the EU. Furthermore, very high groundwater concentrations of this compound have been reported in Asian countries where it is used in rice production to control weeds [9].

The soils formed from sand, which are classified as Arenosols, cover 3.6% of the total land surface of the EU [14]. These are found mainly in Sweden and Poland, as well as in Denmark, northwestern Germany, Lithuania, and Latvia. Subsoils of this group, as well as the subsoils of Luvisols, which cover 14.7% of the EU land surface and are the second most common group of soils in Poland, usually contain a very low content (< 0.4%) of organic matter. The degree of bentazone leaching into groundwater could be higher in these soils than in soils with greater contents of organic matter. To some extent, the adsorption of bentazone on inorganic soil components could prevent its leaching into the groundwater. However, the mechanisms and contribution of this adsorption of bentazone to its total sorption in native mineral soils has not yet been examined.

Therefore, present study aimed to (i) determine the adsorption parameters and assess the contribution of organic and inorganic components of soil to the adsorption of bentazone, and (ii) create a model capable of predicting the adsorption of this compound based on soil properties and the predominant adsorption mechanisms.

## Materials and methods

### Soils

Samples from 11 profiles, classified as Arenosols according to the IUSS Working Group WRB [15], nine profiles classified as Luvisols, and seven profiles classified as Luvisols or Cambisols (indicated henceforth as AR, LV and LV&CM, respectively) were selected from the database and soil collection of the Institute of Agrophysics of the Polish Academy of Sciences in Lublin [16]. AR represents 27% of the coarsest Polish arable soils formed from sand, LV represents 24.7% of the soils formed from loamy sand or loam, and LV&CM represent 6.9% of the soils formed from loess or loess-like formations. The first two letters of the soil acronyms in this study (e.g., AR45Ap or LV913BC) specify the name of the soil group, next is the soil profile number, and the last letters are the symbols of the soil horizon. The profile numbers (from 45 to 913) are the same as in the database of the Institute of Agrophysics [16]. The contents of sand (*C*_*sand*_) in the AR, LV, and LV&CM profiles were 82.2–98.7%, 42.0–86.3%, and 13.0–26.0%, respectively; the contents of clay (*C*_*clay*_) were 0.8–3.1%, 3.5–28.0%, and 2.1–19.7%, respectively; the contents of organic carbon (*C*_*oc*_) were 0–1.42%, 0.06–1.69%, and 0.12–1.80%, respectively; the contents of exchangeable Al extracted in 1M KCl (*C*_*Al*_) were 0.2–62.7, 0–260.1, and 0–43.3 mg/kg, respectively; the contents of exchangeable Fe extracted in 1M KCl (*C*_*Fe*_) were 0.3–4.9, 0.2–5.6, and 0–1.7 mg/kg, respectively; the contents of exchangeable Mn extracted in 1M KCl (*C*_*Mn*_) were 0–83.8, 0.5–134.6, and 0–161.8 mg/kg, respectively; and the pH values in 0.01 M CaCl_2_ were 3.9–6.6, 4.2–6.8, and 4.9–7.7, respectively. The properties of 81 soils analyzed in this study and the methods of analysis are described in S1 Table in S1 Appendix and the other details are described elsewhere [17,18].

### Adsorption experiments at native pH

Aqueous solutions of bentazone, at concentrations of 75, and 7.5 mg/L, were prepared from the certified analytical standard (purity 99.8 ± 0.1%; Institute of Organic Industrial Chemistry, Warsaw, Poland) using sterile redistilled water. The other solvents and reagents used in the study were of analytical or high-performance liquid chromatography (HPLC)-grade.

Batch adsorption experiments were performed according to the OECD Guideline 106 [19] at a temperature of 22±1°C. After selection an appropriate soil–solution ratio (1:1), the analyses on adsorption kinetics were carried out in samples from profiles 611, 590, and 564, which were assumed as the representatives of AR, LV and LV&CM soil groups, respectively. Duplicate samples of soils with a dry weight of 2 g were added to 10 mL glass tubes. Then, 1.735 mL of 0.0115 M CaCl_2_ containing 3·10^−5^ M HgCl_2_ (used as a biocide) was added, and the tubes were equilibrated overnight. Next, 0.265 mL of 7.5 mg/L bentazone was added (initial concentration was 1.0 mg/L), and the tubes were agitated on a rotary shaker for 0.5, 2, 4, 8, 24 or 48 h. The samples were centrifuged (10 min, 3300 g, 20°C), and the liquid phase was separated for analyses. The results of kinetic experiments indicated that the adsorption equilibrium was attained at a time of ≤ 8 h (S4 Fig and S6 Table in S4 Appendix). Therefore, a duration of 24 h was assumed as sufficient to attain adsorption equilibrium.

The same conditions of adsorption were applied to experiments conducted for *K*_*d*_ determination in 81 soils (S1 Table in S1 Appendix), and in native AR611C fractions of 2000–500, 500–400, 400–150, 160–63, 63–40, and < 40 μm, obtained by sieving the soil (30 min) using a Retsch AS200 vibratory sieve shaker (S11 Table in S8 Appendix). In each of the duplicate samples, the initial concentration of bentazone was 1.0 mg/L. After 24 h of shaking, the pH of the samples was measured using a glass electrode. The samples were centrifuged, and the liquid phase was collected for the HPLC analysis. The amounts of adsorbed bentazone were calculated based on the difference between the initial and the final concentrations of the substance in solution.

### pH-dependent adsorption experiments

These experiments were carried out in the selected soils (AR232C, AR360C, AR774C, and AR774C in which the oxides and hydroxides of Fe and Al were removed by using the method of Mehra and Jackson [20]). Briefly, duplicate soil samples of 2 g dry weight were added into 10 mL glass tubes, to which 1.735 mL of redistilled water was added. Then, small portions of 0.1 M HCl or 0.1 M NaOH were added over 12 h. In this way, the required equilibrium pH was achieved in the soil suspensions. The excess solution was removed after centrifugation (10 min, 3300 g, 20°C) to obtain a liquid volume of 1.700 mL. To this, 0.035 mL of a solution containing 0.57 M CaCl_2_ and 1.5·10^−3^ M HgCl_2_ was added. Then, 0.265 mL of 7.5 mg/L bentazone solution was added (final concentration was 1.0 mg/L), and the tubes were agitated for 24 h. The next steps of the experiments were the same as those conducted for the determination of *K*_*d*_ values.

The above procedure was slightly modified for the adsorption experiments on AR774C samples sieved through a 500 μm mesh, designed for the Fourier transform infrared photoacoustic spectroscopy (FT-IR/PAS) measurements. Duplicate AR774C samples (fraction < 500 μm) weighing 2 g were added into 10 mL glass tubes, to which 1.735 mL of redistilled water was added. The pH of the suspension was adjusted to desired levels by adding 0.1 M HCl or 0.1 M NaOH over 12 h. After removing the excess solution following centrifugation (10 min, 3300 g, 20°C) to obtain the volume of 1.735 mL, 0.265 mL of 75 mg/L bentazone was added (in this case final concentration was 10.0 mg/L). Then, the tubes were agitated on a rotary shaker for 24 h, and the pH of the samples was measured. The suspensions were transferred to 25 mL, centrifugal filters (0.45 μm polyvinylidene fluoride (PVDF) membrane, Thermo Scientific^™^) and centrifuged (15 min, 2500 g, 20°C). The solutions were collected for the HPLC analyses, and the AR774C samples selected for the FT-IR analyses were dried at 35°C for 48 h.

### Analytical methods

#### HPLC analyses

A 30 μl portion of bentazone solution was injected into a Waters HPLC equipped with a Waters 600 quaternary pump, Waters In-Line Degasser AF, Waters 2998 Photodiode Array Detector (DAD), Waters 2707 Autosampler, and Waters 600 Controller with the Empower2 software. A Knauer Eurosil Bioselect-300 C_18_ column (300 × 4.0 mm, 5 μm particle size) was used and temperature was maintained at 35°C using a Varian PCB 150 Water Peltier System. The mobile phase was 3.8% acetonitrile/100 mM/L acetate buffer with pH 5.3 (90:10 v/v). The flow rate of the mobile phase was 1.6 mL/min, the run time was 8.5 min per sample, and the detection wavelength was 220 nm. All measurements were performed in triplicate. The detection limit was 0.05 mg/L, and the relative standard deviation of the repeatability of results < 1%.

#### Microscopic analyses

Micro-area analyses were performed using a Hitachi SU6600 scanning electron microscope attached with a Thermo Scientific EDS. The representative soil samples weighing ≤ 1g were placed using a spatula under the binocular magnifier on a carbon tape partitioned with paper. Then, the samples were transferred to the electron microscope chamber, and analyses were carried out under a low vacuum (about 10 Pa) [21]. Samples were examined with a 15 kV beam for 60 s. Each grain of the soil sample containing about 150 grains was tested separately. The elemental composition of the samples was determined by irradiating each grain for the same time. Results are presented in S2 Fig in S1 Appendix, and S6 and S7 Figs in S8 Appendix. These studies were carried out at the Department of Geology, Soil Science and Geoinformation of the Maria Curie-Skłodowska University in Lublin.

#### X-ray diffraction analyses

The mineralogical composition of the selected soils was determined using ground samples (30 min, 28 Hz; Retsch MM200 Oscillating Mill Grinder) by applying the powder X-ray diffraction technique (Empyrean, Malvern Panalytical diffractometer with a Cu anode used as a source of CuKα X-ray radiation (λ = 1.5406 Å)). The diffraction data were fitted using the ReX v.0.91 Rietveld analysis software [22]. For analysis, the necessary crystal structure files (cif) were downloaded from the American Mineralogist Crystal Structure Database [23], based on the results obtained from the microscopic and elemental analyses of soil grains. More details of this analysis can be found in S3 Appendix.

#### IR analyses

FT-IR/PAS spectra of the selected soil samples with adsorbed bentazone were recorded using a Bio-Rad Excalibur FT-IR 3000 MX spectrometer over a 3400–900 cm^–1^ range at room temperature, with a resolution of 4 cm^–1^ and maximum source aperture, using a MTEC Model 300 photoacoustic cell. Dry helium was used to purge the photoacoustic cell before data collection. The spectra were normalized by computing the ratio of a sample spectrum to the spectrum a MTEC carbon black standard. A stainless steel cup (diameter 10 mm) was filled with sample (thickness < 6 mm), and the interferograms of 1024 scans were averaged for the spectrum, providing a good signal-to-noise (S/N) ratio. All spectral measurements were performed at least in triplicate.

#### *pK*_*a*_ determination

The *pK*_*a*_ of bentazone (*pK*_*a*_ = -log*K*_*a*_, *K*_*a*_ is the dissociation constant) was determined experimentally using the spectrophotometric method [24], as described in S2 Appendix. The value of *pK*_*a*_ obtained at a temperature of 20°C and ionic strength of 0.01 was 2.86, with a standard estimation error of 0.01. The value of *pK*_*a*_^*T*^, which is the thermodynamic dissociation constant independent of the concentration [24], was 2.91. This value is close to the value *pK*_*a*_^*T*^ = 2.92 ± 0.06 obtained at 20°C by Comer et al. [25] by applying the pH-metric technique. These measurements were carried out in this study due to the reason that a wide range of values, including 2.03, 2.50, 3.20, 3.30, and 3.51 are found in the online databases and literature sources [1,4,11,26,27].

#### PZNPC determination

The point of zero net proton charge (PZNPC) of the AR774C soil was determined using the potentiometric titration method as described by Schroth and Sposito [28] and Pansu and Gautheyrou [29]. Briefly, 2 g samples of < 500 μm fraction were added to 30 mL polypropylene tubes and 20 mL of the respective solution was dosed. The pH values of the prepared solutions were adjusted using 0.01 M HCl or NaOH, while their ionic strength was adjusted by adding NaCl as indifferent electrolyte. Three series of samples having NaCl at concentrations of 0.0025, 0.05, and 0.15 M were prepared. The screwed tubes containing the soil and the blank samples (tubes with solution but no soil) were agitated for 48 h. Then, the pH of each soil sample and the respective blank sample was measured. A detailed description of the experiment is provided in S7 Appendix.

#### Removal of Al and Fe oxides and hydroxides

For some adsorption experiments, soil samples in which the oxides and hydroxides of Al and Fe were removed with the dithionite-citrate-bicarbonate method [20] were used. The Al and Fe oxides and hydroxides were also extracted using the Tamm’s solution (0.2 M oxalic acid and ammonium oxalate solution adjusted to pH 3.0) [29]. A detailed description of the used procedures can be found in S1 Appendix.

### Modeling of adsorption in soils

Usually, both anionic and neutral forms of acidic pesticides are adsorbed on the organic matter present in soil. The following equation describes this process of pH-dependent adsorption [18,30–32]:

$$K_{d}=\kappa_{oc(n)}\cdot C_{oc}\cdot \Phi_{n}{+\kappa_{oc(an)}\cdot C_{oc}\cdot \Phi }_{an}$$ (1)

where *ĸ*_*oc(n)*_ is the adsorption coefficient at the pH at which the pesticide molecules are not dissociated and adsorption of the neutral form is at the maximum, and *ĸ*_*oc(an)*_ is the adsorption coefficient at the pH at which the pesticide molecules are dissociated and adsorption of the anionic form reaches its maximum. The fractions of the neutral (Φ_*n*_) and anionic (Φ_*an*_) forms of the pesticide are calculated using the rearranged equation for the dissociation constant of a weak monovalent acid as follows:

Φn=1(1+10pH−pKa) (2)

$$\Phi_{an}=1-\Phi_{n}$$ (3)

For the adsorption of a pesticide that is capable of dissociating in the pH range of the examined soils, pH is identified as the variable highly affecting the process. The relationship between *K*_*d*_ and the content of soil components contributing to adsorption is nonlinear (e.g., the relationship between *K*_*d*_ and *C*_*oc*_ shown in Eq (1)). This is due to the fact that the variance of pH of the soil–water system entails a nonlinear change in the ratio of molecular and ionized forms of pesticide, which results in nonlinear adsorption.

However, the pH variable can be eliminated and the nonlinear relationships can be linearized. The data obtained from batch experiments include pairs of *K*_*d*_ and pH values, and it is easy to calculate the values *Φ*_*n*_ and Φ_*an*_ for each soil sample. Therefore, the transformed variables can be created by multiplying the variable *C*_*x*_ assumed to be involved in pesticide adsorption with the calculated values of *Φ*_*n*_ or *Φ*_*an*_. In this study, for the initial analysis of the relationships between *K*_*d*_, pH and the *C*_*oc*_, *C*_*sand*_, *C*_*silt*_, *C*_*clay*_, *C*_*Al*_, *C*_*Fe*_ and *C*_*Mn*_ variables the analysis based on Kendall’s rank correlation (denoted as *r*_*K*_) was used. Next, the matrices of the transformed variables were created, suspecting the adsorption of neutral (*C*_*oc*_*Φ*_*n*_, *C*_*sand*_*Φ*_*n*_, *C*_*silt*_*Φ*_*n*_, *C*_*clay*_*Φ*_*n*_, *C*_*Al*_*Φ*_*n*_, *C*_*Fe*_*Φ*_*n*_, and *C*_*Mn*_*Φ*_*n*_) and anionic (*C*_*oc*_*Φ*_*an*_, *C*_*sand*_*Φ*_*an*_, *C*_*silt*_*Φ*_*an*_, *C*_*clay*_*Φ*_*an*_, *C*_*Al*_*Φ*_*an*_, *C*_*Fe*_*Φ*_*an*_, and *C*_*Mn*_*Φ*_*an*_) forms of bentazone. It is worth noting that, according to Eq (1), the relationships between *K*_*d*_ and the transformed variables *C*_*oc*_*Φ*_*n*_ and *C*_*oc*_*Φ*_*an*_ (and any other *C*_*x*_*Φ*_*n*_ and *C*_*x*_*Φ*_*an*_ variables) should be linear because the coefficients *ĸ*_*oc(n)*_ and *ĸ*_*oc(an)*_ are estimated for pH ranges at which the adsorption process is independent of pH (explained by the definitions of *ĸ*_*oc(n)*_ and *ĸ*_*oc(an)*_). The main disadvantage of this transformation is the increase of collinearity between the independent variables. For these reasons, the exploratory analysis of the transformed variables was performed using the partial least squares regression (PLSR) analysis.

In the PLSR analysis [33,34], the determined *K*_*d*_ values were used as Y-variable and the transformed variables as X-variables (similar to the study of Paszko [18]). The number of latent vectors for each dataset was estimated for the data cross-validated and partitioned into five blocks, assuming that Wold’s *Q*^2^ must exceed 0.0975. The significant variables during the PLSR analysis were selected by the stepwise backward elimination of X-variables with the smallest values of the standardized coefficient and the values of variable importance on projection (VIP). It was assumed that VIP > 1 yields the most relevant variables, a range of 0.8–1.0 indicates a moderate influence, and < 0.8 characterizes the least important variables.

The PLSR analysis was carried out with the XLSTAT 2018.7 software [35], the nonlinear weighted regression with DataFit 9.1 (Oakdale Engineering), and correlation analysis with Statgraphics Centurion XVII (Statpoint Technologies, Inc.).

The model of a bentazone molecule was built with a standard bond length and angles using the PC SPARATN’10 Pro Ver. 1.1.0 molecular modelling program [36]. The energy was minimized by the molecular mechanical methods, and the lowest energy conformer was further optimized. The applied models were: semi empirical PM3, Hartree–Fock RHF 6–311+G**, and STO-3G, density functional theory DFT/B3LYP/6-311++G(d,p), and ground-state equilibrium geometry. The PM3 (parametric method 3) is a semi-empirical more advanced method based on the Neglect of Differential Diatomic Overlap (NDDO) and on the empirical parameters as optimizable values. The *ab initio* 6–311+G** basis set is a valence triple-zeta polarized basis set that adds a set of polarizing d-functions on heavy atoms and a set of polarization p-functions on hydrogen (6-311G(d,p)) [37]. For the optimization of a molecule in water the RHF STO-3G bases set was applied. The charge distribution in the molecule was calculated by the Hartree–Fock method at 6-311G** level. This bases set is often used to calculate the electronic properties of small organic molecules and their biological activity [38]. The charge of atoms from the electrostatic potential distribution as well as the Mullicen charge were determined according to Singh and Kollman [39].

## Results and discussion

### Relationship between adsorption and soil properties–results of correlation analysis

**Fig 1:**
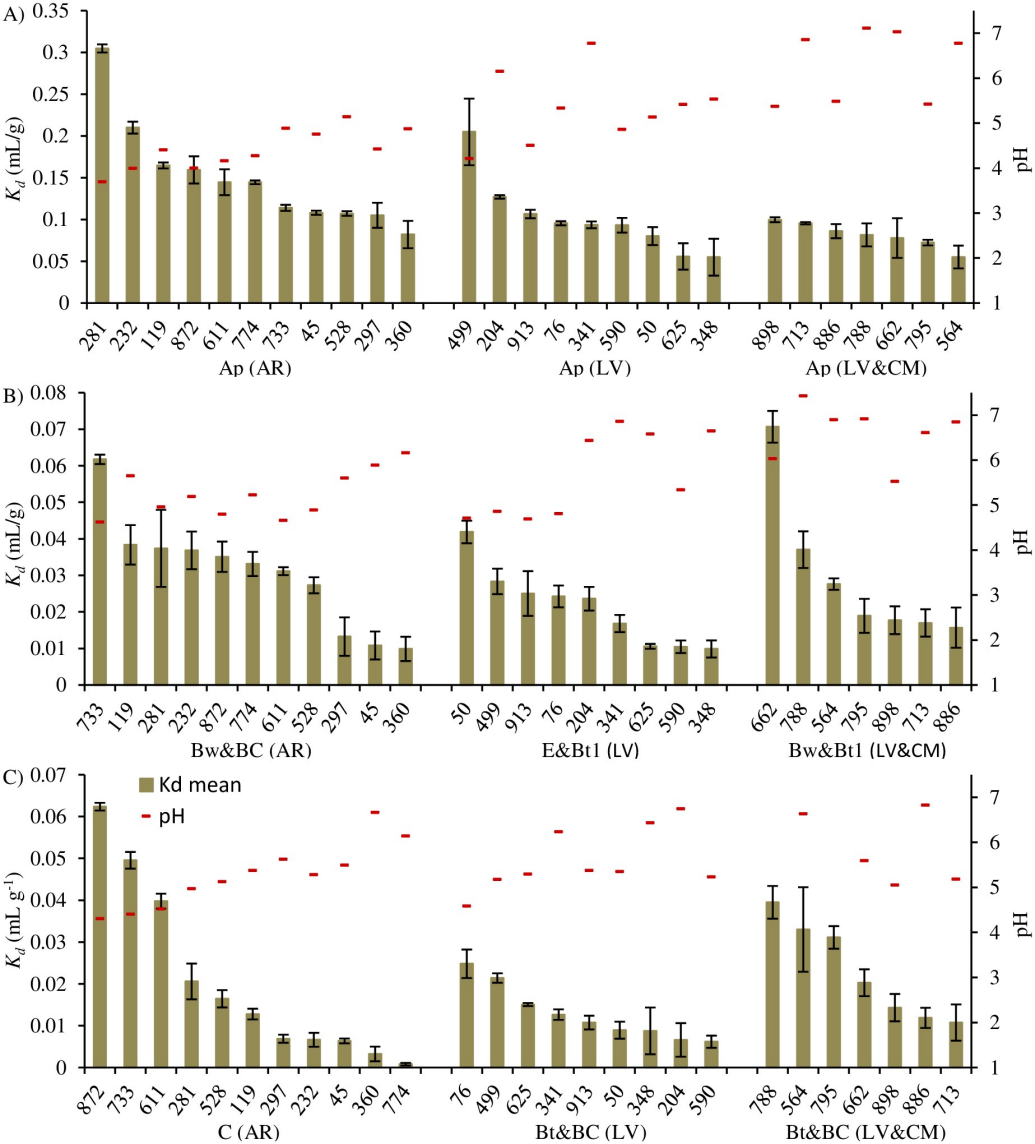
Values of *K*_*d*_ and pH in the soil suspensions in the topsoil (A) and subsoils (B-C) of the examined Arenosol (AR), Luvisol (LV), and Luvisol and Cambisol (LV&CM) profiles. Error bars denote ±SD.

The *K*_*d*_ values determined for the 81 soils were arranged in decreasing order for each of the examined soil groups and are presented in Fig 1. The highest degree of adsorption with a mean *K*_*d*_ value of 0.15 mL/g was observed in topsoils of the AR soil group (Fig 1A), while lower adsorption was noted in topsoils of the LV and LV&CM soil groups with mean *K*_*d*_ values of 0.10 and 0.08 mL/g, respectively. The greatest adsorption in the Ap horizon of AR can be attributed to the highest acidity of soils in this group (mean pH of AR was 4.4, LV was 5.3, and LV&CM was 6.0), which affected adsorption to a larger extent than the content of organic carbon (mean values were 1.04%, 1.15%, and 1.30%, respectively). The variance of the *K*_*d*_ values in subsoils (Fig 1B and 1C) also combined primarily with the differences in pH and the content of organic matter. Fig 1C shows the high adsorption in samples from the lower subsoil horizon of profiles 872, 733, and 611 of AR, which suggests that these results stand out from others. For example, adsorption in these samples was much higher than in the samples of profile 76 of LV. Taking into account the pH and *C*_*oc*_ contents in these four subsoils (range of 4.3–4.5 and 0.03–0.11%, respectively; S1 Table in S1 Appendix), it was suspected that organic matter was not the only soil component that adsorbed bentazone. It worth noting that the *C*_*sand*_ content was in these subsoils high–in the range of 94.3–98.0% in the AR profiles and 59.3% in the LV profile, which suggested its potential contribution in adsorption.

The Shapiro-Wilk test performed for the determined *K*_*d*_ values and the examined soil properties (pH, *C*_*oc*_, *C*_*sand*_, *C*_*silt*_, *C*_*clay*_, *C*_*Al*_, *C*_*Fe*_, and *C*_*Mn*_) showed that the variables did not follow the normal distribution (*p* < 0.05). One reason for this observation was that the soils were selected based on their inclusion in the specific soil groups, which differentiated their properties. A second reason was that samples from the individual horizons of soil profiles were examined in the study. From a statistical point of view, samples from different soil groups or horizons may be treated as belonging to different populations. Hence, for the initial analyses the Kendall rank correlations were calculated. The correlation analysis indicated (S8 Table in S5 Appendix) that the increase in *K*_*d*_ was consistent with the increase of *C*_*oc*_ (*r*_*K*_ = 0.644, *p* < 0.001), and to a much lesser extent, the increase of *C*_*Mn*_, *C*_*Fe*_, and *C*_*Al*_ (*r*_*K*_ = 0.332, 0.307, and 0.293, respectively; *p* < 0.001). However, *C*_*oc*_ was positively correlated with Mn cations (*r*_*K*_ = 0.380, *p* < 0.001) and Fe cations (*r*_*K*_ = 0.198, *p* = 0.009). An inverse correlation was observed between *K*_*d*_ values and pH (*r*_*K*_ = -0.306, *p* < 0.001) as well as between *K*_*d*_ values and *C*_*clay*_ (*r*_*K*_ = -0.178, *p* = 0.019). Thus, the simple correlation analysis could not clearly explain which soil component, except organic matter, was involved in bentazone adsorption.

Positive correlation between the *K*_*d*_ and organic matter content and the inverse between *K*_*d*_ and pH was observed earlier by Li et al. [4], and Rodríguez-Cruz et al. [40]. The *K*_*d*_ values obtained by Madsen et al. [41] were inversely related to pH, positively to *C*_*sand*_, and no correlation was observed between *K*_*d*_ and *C*_oc,_ as well as with oxalate extracted Al, and Fe oxides and hydroxides. Positive correlation between *K*_*d*_ and *C*_*sand*_ was obtained also by Li et al. [4]. In this study the correlation between *K*_*d*_ and *C*_*sand*_ for the whole dataset of 81 soils was insignificant (S8 Table in S5 Appendix).

### Modeling the pH-dependent adsorption of bentazone

One of the best regression methods that tolerate noise in variables and cope with collinearity is PLSR [33,34,42]. Therefore, it was applied for the exploratory data analysis. In the first step of PLSR, all the 14 examined variables were used. The optimal number of latent factors for this variable matrix was 2. The analysis explained the 93.73% of the *K*_*d*_ variance (Table 1) and indicated that five variables (*C*_*clay*_*Φ*_*n*_, *C*_*sand*_*Φ*_*an*_, *C*_*silt*_*Φ*_*an*_, *C*_*clay*_*Φ*_*an*_, and *C*_*Al*_*Φ*_*an*_) were not significant for predicting *K*_*d*_ values. These variables had a negative standardized regression coefficient and a VIP value of ≤ 0.8. After reducing the number of variables to 9, the model explained 93.60% of the *K*_*d*_ variance. Similarly in the next steps, variables such as *C*_*silt*_*Φ*_*n*_, *C*_*Al*_*Φ*_*n*_, *C*_*Fe*_*Φ*_*n*_, *C*_*Mn*_*Φ*_*n*_, *C*_*Fe*_*Φ*_*an*_, and *C*_*Mn*_*Φ*_*an*_ were considered insignificant. After 5 more steps, the number of variables was further reduced to 3. Nevertheless, the model explained 96.46% of the *K*_*d*_ variance– 2.73% more than the initial 14-variable model and only 0.23% less than the five-variable model that explained most of the *K*_*d*_ variance. Therefore, it was assumed that variables other than *C*_*oc*_*Φ*_*n*_, *C*_*sand*_*Φ*_*n*_, and *C*_*oc*_*Φ*_*an*_ are insignificant. On the other hand, removing the *C*_*sand*_*Φ*_*n*_ variable reduced the *R*^*2*^ value by 2.32% (Table 1), which is also only a slight difference. However, it should be remembered that only 27 out of the 81 tested soils had a pH < 5, and only in such soils of the neutral form of bentazone might adsorb on the sand surface. The final model obtained using PLSR was:

$$K_{d}=\;0.005\;+\;1.099\cdot C_{oc}\Phi_{n}+\;0.065\cdot C_{oc}\Phi_{an}+\;0.010\cdot C_{sand}\Phi_{n}$$ (4)

**Table 1:** Results of PLSR analysis.

	Step 1	Step 2	Step 3	Step 4	Step 5	Step 6	Step 7
LF^a^	2	2	2	2	2	2	1
*R*^*2*^*Y cum*	93.73	93.60	95.9s9	96.69	96.55	96.46	94.14
*N*	81	81	81	81	81	81	81
*C*_*oc*_*Φ*_*n*_	0.165^1.3^^b^	0.160^1.1^	0.166^1.1^	0.249^1.1^	0.296^1.1^	0.378^1.1^	0.639^1.1^
*C*_*sand*_*Φ*_*n*_	0.130^1.2^	0.127^1.0^	0.121^1.0^	0.206^1.0^	0.243^1.0^	0.318^1.0^	
*C*_*silt*_*Φ*_*n*_	0.093^1.1^	0.071^1.0^	0.069^0.9^				
*C*_*clay*_*Φ*_*n*_	-0.018^0.8^						
*C*_*Al*_*Φ*_*n*_	0.080^1.1^	0.061^1.0^	0.043^0.9^				
*C*_*Fe*_*Φ*_*n*_	0.110^1.2^	0.089^1.0^	0.076^0.9^				
*C*_*Mn*_*Φ*_*n*_	0.157^1.2^	0.164^1.0^	0.198^0.9^	0.186^0.9^	0.203^0.9^		
*C*_*oc*_*Φ*_*an*_	0.321^1.5^	0.399^1.3^	0.498^1.3^	0.504^1.1^	0.519^1.0^	0.549^1.0^	0.549^0.9^
*C*_*sand*_*Φ*_*an*_	-0.023^0.3^						
*C*_*silt*_*Φ*_*an*_	-0.050^0.4^						
*C*_*clay*_*Φ*_*an*_	-0.128^0.6^						
_*Al*_*Φ*_*an*_	-0.048^0.6^						
*C*_*Fe*_*Φ*_*an*_	0.125^1.1^	0.126^0.9^	0.132^0.8^	0.143^0.9^			
*C*_*Mn*_*Φ*_*an*_	0.129^0.7^	0.148^0.5^					

^a^LF = number of latent factors.

^b^Standardized regression coefficient^VIP value^.

The intercept value in Eq (4) was very low; therefore, a *p*-value of > 0.05 appeared likely for this coefficient. The high variance of *K*_*d*_ in some soils, especially in the Ap horizon (Fig 1), in turn suggested use of weighted regression. For this reason, nonlinear weighted regression was performed for the final model, with standard deviations of *K*_*d*_ used as weights. The equation obtained was:

$$K_{d}=\;0.226\cdot C_{oc}\cdot \Phi_{n(p=0.012)}+\;0.072\cdot C_{oc}\cdot \Phi_{an(p<0.001)}+\;0.018\cdot C_{sand}\cdot \Phi_{n(p<0.001)}\;R^{2}=0.985$$ (5)

**Fig 2:**
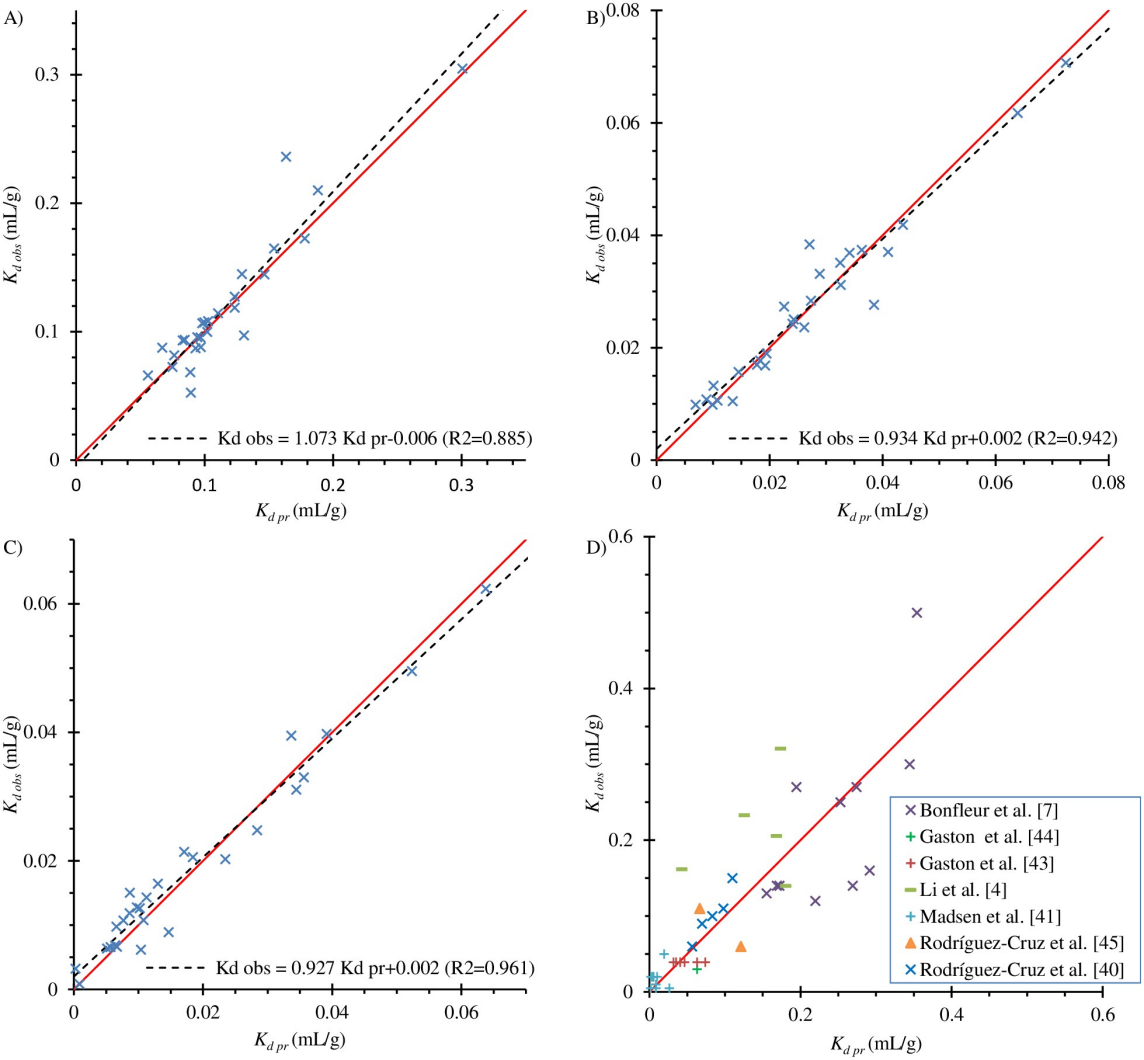
Observed versus predicted using Eq (5) *K*_*d*_ values for the examined topsoil (A), upper (B), and lower subsoil horizons (C) as well as *K*_*d*_ values observed by the other authors versus the *K*_*d*_ values predicted using Eq (5) (D).

The *p*-values calculated for the Shapiro-Wilk normality tests of residuals examining the H_0_ hypothesis were 0.019, 0.606, and 0.323 for topsoil and two subsoils, respectively. Despite the lack of normal residuals distribution in the Ap horizon, the use of weighted regression allowed to correctly estimate the regression coefficients in Eq (5) (Fig 2A–2C). In each of the examined soil levels, the regression lines that expressed the relationships between the predicted and observed *K*_*d*_ values only differed slightly from the curve Y = X. The obtained for Eq (5) *R*^*2*^ value (0.985) is very high, but the one obtained using the weighted least squares method is higher than that calculated from the ordinary least squares; the greater the difference, the greater the weights are. In fact, the model explained 96.1% variance of the experimental *K*_*d*_ values.

Fig 2D shows the relationship between the *K*_*d*_ values obtained by other authors and the values predicted using Eq (5). It is noticeable that the *K*_*d*_ values obtained by Bonfleur et al. [7], Gaston et al. [43] and [44], Madsen et al. [41], and Rodríguez-Cruz et al. [45] and [40] lie along the line Y = X. Thus, the adsorption coefficients determined using Eq (5) predicted the sorption properties of these European, North American, and South American soils as well. The *K*_*d*_ values determined for Chinese soils by Li et al. [4] are slightly shifted to the left of the line Y = X. In their study the authors conducted the adsorption experiments in 0.01 M CaCl_2_ but provided the pH values of soils measured in H_2_O (S9 Table in S6 Appendix), which are higher. Therefore, the *K*_*d*_ values calculated using Eq (5) were underestimated. Thus, the proposed model correctly predicted *K*_*d*_ values for the soils described in the available literature. The results presented in Fig 2D show that the affinity organic matter and sand to bentazone is surprisingly constant, even though the soils came from different geographical regions of a given country, or even from different continents.

Eq (5) is consistent with the model assuming adsorption of bentazone on organic matter obtained for five topsoils with *C*_*oc*_ in the range of 0.37–2.49% by Li et al. [4]. Taking into account results presented in Fig 2B and 2C, it enable correct prediction of adsorption also for soils with *C*_*oc*_ < 0.37%. As shown in Fig 2D the sorption properties of Brazilian tropical and subtropical Oxisols [7] were also similar to those of other soils. Oxisols are characterized by the large contents of Al and Fe oxides and hydroxides. Therefore, the data obtained by the authors suggested that in addition to adsorption in organic matter, bentazone was in these soils absorbed also by Al oxides.

### Adsorption in soils containing no organic matter

**Fig 3:**
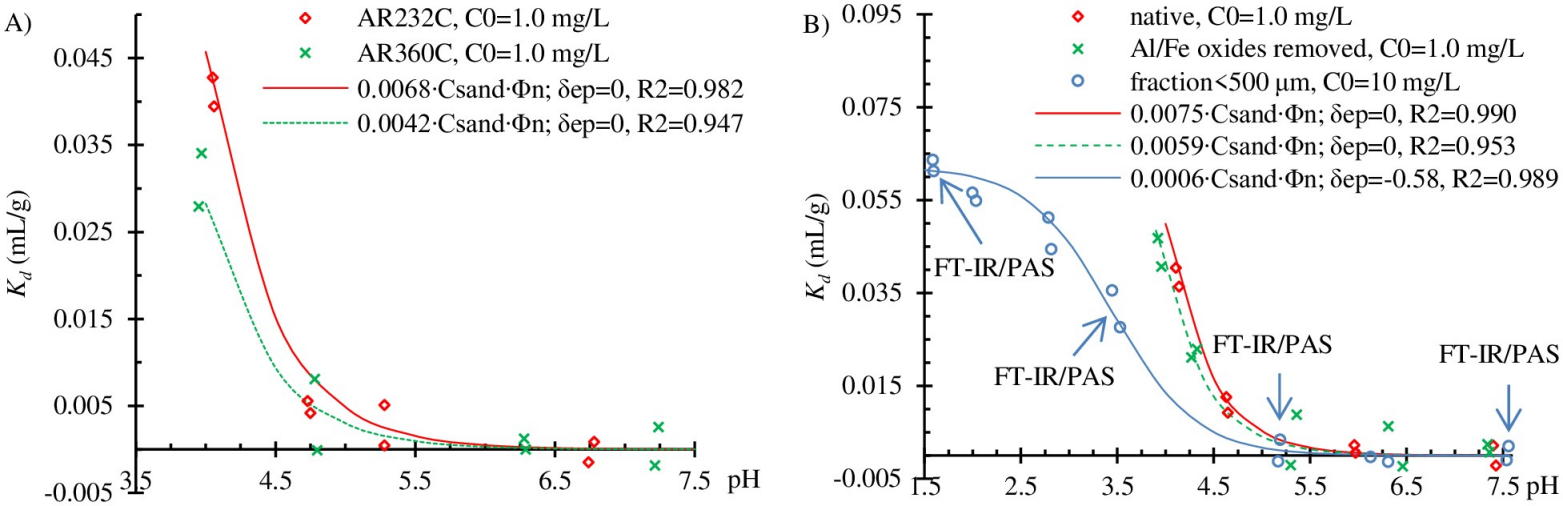
Observed *K*_*d*_ values and results of modeling of the pH-dependent adsorption in soils with no organic matter. (A) AR232C and AR360C, (B) AR774C, AR774C with removed Al and Fe oxides [20], as well as in AR774C fraction < 500 μm with ten times higher initial bentazone concentration.

Samples of three of the analyzed AR from the C horizon (AR232C, AR360C, and AR774C) practically contained no organic matter. Considering the detection limit of the TOC-VCSH analyzer used, the *C*_*oc*_ content in these three soils was < 0.0005%. Therefore, it was assumed that bentazone adsorption of by such a low content of organic matter should be negligible, and the soils were used to determine the possible extent of bentazone adsorption on inorganic soil components. The results showed that there was no adsorption in the soils at pH > 5.5 (Fig 3A and 3B). However, at pH < 5, the adsorption was noticeable. The obtained *K*_*d*_ values were in the same range as that (0.026 ± 0.007 mL/g) determined by Clausen et al. [8] for the adsorption of bentazone on quartz at pH 2.4 and 10°C (the value was converted from unit mL/m^2^ used by the authors). The shape of the pH-dependent adsorption curves observed in Fig 3A and 3B is similar to that for bentazone adsorption on mesoporous silica as observed by Bruzzoniti et al. [9]. The only difference is that Bruzzoniti et al. [9] (similarly to Spaltro et al. [10]) observed also weak bentazone adsorption in the pH range of 5–7. The total specific surface area of silica (SSA) determined from the BET N_2_ isotherms examined by Spaltro et al. [10] was 264 m^2^/g and SSA of silica examined by Bruzzoniti et al. [9] was in the range of 484–876 m^2^/g. The SSA determined from the N_2_ adsorption isotherms of subsoils of Polish AR is typically in the range of 0.7–2.6 m^2^/g ([46], see the data for subsoils of 611 and 805 profiles). Thus, the most likely reason why in Fig 3A and 3B adsorption in the pH range of 5–7 was not observed is that the number of active in this pH sorption sites was too small in the examined soils. It is worth noting that also Clausen et al. [8] did not observe adsorption of bentazone on quartz with SSA 0.83 m^2^/g at pH 6.5.

According to Clausen and Fabricius [11], at pH ≤ 5.5, bentazone can be adsorbed by Fe oxides, and the extent of adsorption should be much higher than that by quartz [8]. Therefore, Fe and Al oxides and hydroxides were removed from the AR774C samples according to the dithionite-citrate-bicarbonate method [20]. As seen in Fig 3B, in the altered soil samples, there was a slightly lower adsorption compared to the native soil. However, this difference was too low to be significant. Thus, the result of the last experiments suggested that adsorption at pH < 5 occurs predominantly on inorganic soil components other than the Fe and Al oxides and hydroxides.

Fig 3B also shows the results of the adsorption experiment conducted on samples of the AR774C fraction sieved through a 500 μm sieve. In this experiment, the initial concentration of bentazone was increased to 10 mg/L to obtain sufficient adsorbed amounts for the FT-IR/PAS analysis. It was found that the decrease in the soil suspension pH to 1.6 increased the *K*_*d*_ values to 0.061 mL/g. The pH of PZNPC of the AR774C fraction < 500 μm was 5.15 (S5 Fig in S7 Appendix). Thus, in the range of pH < 5, at which bentazone adsorption was observed, the soil surface was positively charged. Clausen et al. [8] suggested that the positively charged surfaces of quartz can adsorb bentazone anions. The point of zero charge of pure quartz is in the range of 2.2–2.9 [47,48], but in sands, higher values of up to value 6.0 were observed [49]. The shape of the pH-dependent adsorption curve shown in Fig 3A and 3B suggested that the neutral form of bentazone was adsorbed rather than the anionic form.

Therefore, it was hypothesized the neutral form of bentazone was bound by the silanol groups of quartz. When pH decreased below the value of 5, the amount of the neutral form of bentazone increased, but below the soil’s PZNPC the number of silanol groups on quartz surfaces also decreased. Assuming that the concentration of the neutral bentazone form was the limiting factor of adsorption, the mathematical description of such a process can be given as follows:

Kd=κX(n)⋅CX(1+10pH+δep−pKa) (6)

where κ_*X(n)*_ is the adsorption coefficient of the neutral form of bentazone on the X soil component whose concentration is expressed as *C*_*X*_ (in the above case X was sand). *δ*_*ep*_ is the enhanced protonation coefficient, which describes the difference between the pH measured in a soil suspension and that on the soil surface (which is lower). This coefficient has been used by many authors for modeling adsorption of ionizable pesticides (see, e.g., Franco et al. [50]). As seen in Fig 3A, Eq (6) fitted the experimental *K*_*d*_ values sufficiently well for AR232C and AR360C samples, as well as the *K*_*d*_ values for AR774C, and AR774C samples in which the Fe and Al oxides were removed using the method of Mehra and Jackson [20] (Fig 3B), despite setting *δ*_*ep*_ = 0 while fitting.

The results obtained for fitting *K*_*d*_ values for the AR774C fraction < 500 μm, assuming *δ*_*ep*_ = 0, were much worse (*R*^*2*^ = 0.912) compared to when *δ*_*ep*_ was used as a second estimated coefficient (*R*^*2*^ = 0.989). The adsorption curve was of sigmoidal shape due to the wider range of the examined pH. Because soils exhibit buffering abilities, the pH of the soil suspension can only be lowered by the addition of much larger amounts of H^+^ than that required for changing the pH in a bulk solution. The majority of added H^+^ is adsorbed on the soil surface. Thus, the lower is the pH in the soil suspension the greater is the difference between the pH in the bulk solution and that at the soil surface. In the case of bentazone the amount of its neutral form near the soil surface was higher than that in the bulk solution, which increased adsorption. On the other hand, the necessity of using the *δ*_*ep*_ coefficient for adsorption data covering very low pH values could have also resulted from the decrease in the concentration of ≡ SiOH surface groups, and the increase in the concentration of ≡ SiOH_2_^+^ surface groups, or from different adsorption mechanism at very low pH.

It is noticeable that adsorption in the < 500 μm samples of AR774C was much lower than that in the whole AR774C soil. The *K*_*d*_ value estimated at pH 4.0 for AR774C was 0.050 mL/g, while it was only 0.014 mL/g for the fraction < 500 μm (Fig 3B). Because adsorption of bentazone is usually inversely correlated to its concentration (*1/n* < 1), it seems that the principal reason for obtaining lower *K*_*d*_ values for the AR774C fraction < 500 μm was the ten-fold higher initial concentration of bentazone. The reason was the low number of active sites capable of binding bentazone in the tested soil.

### Adsorption in soils with a low content of organic matter

**Fig 4:**
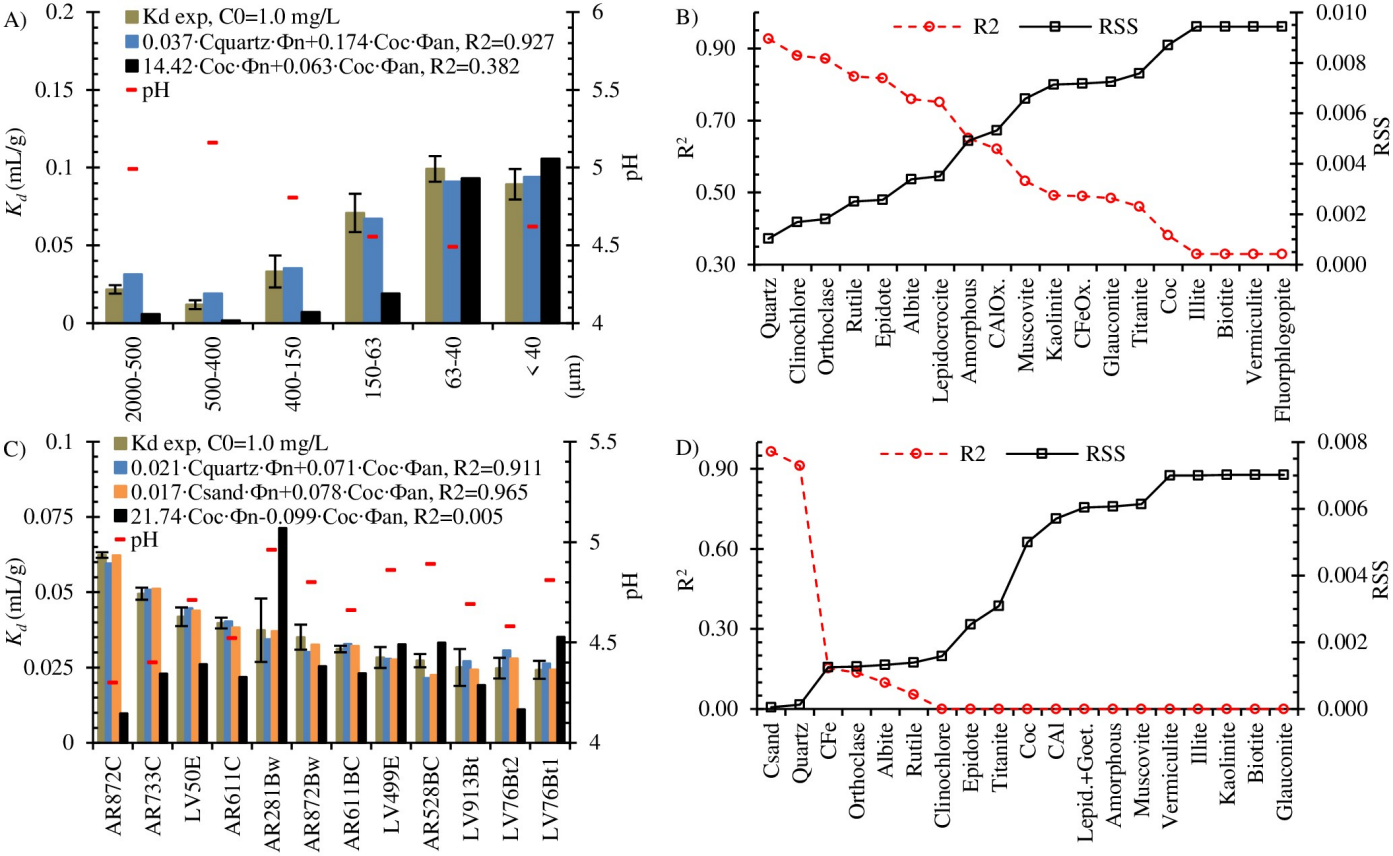
Results of modeling of adsorption in fractions of AR611C (A and B), and in 12 selected soils (pH < 5 and *C*_*oc*_ < 0.35%) (C and D). The *R*^*2*^ and *RSS* (residual sum of squares) values (C and D) are the results of Eq (1) fitting, with the assumption that bentazone anions are adsorbed on organic matter and one of soil components listed on X-axis for adsorption of bentazone molecules.

Fig 4A shows the results obtained for the adsorption experiments conducted on the fractions of AR611C soil with a native pH and organic matter content. The soil was characterized by a very low *C*_*oc*_ (0.03%) and pH in 0.01 M CaCl_2_ (4.5, soil–solution ratio 1:1) and high sand content (96.1%), and was one of the subsoils that showed the highest adsorption (*K*_*d*_ = 0.040 mL/g, S7 Table in S4 Appendix). The basic physical–chemical properties of these fractions and their mineralogical composition are presented in S11 Table in S8 Appendix. It is surprising that noticeable adsorption occurred even in the fractions of 2000–500 and 400–150 μm with *C*_*oc*_ = 0.03%, while high adsorption was observed in the fraction of 150–63 μm with *C*_*oc*_ = 0.05%. The *C*_*oc*_ contents in the fractions 63–40 and < 40 μm were 0.24% and 0.34%, respectively.

The adsorption data were analyzed using Eq (1) with an assumption that organic matter is responsible for the adsorption of bentazone anions, while one of the 19 soil components (including organic matter) listed in S11 Table in S7 Appendix is specifically responsible for the adsorption of its neutral form. The experiment was carried out in the pH range of 4.49–5.16 (S10 Table in S7 Appendix), which allowed analyzing the adsorption of the neutral form of bentazone. The fitting results indicated that the principal variable associated with the adsorption of bentazone molecules was quartz. Its content was the lowest (56.2%) in the fraction < 40 μm and highest (93.3%) in the fraction 2000–500 μm. According to the results presented in Fig 4B it cannot be ruled out that to some extent adsorption occurred on clinochlore and orthoclase. However, the amounts of these minerals were much lower in comparison to quartz. The nonlinear form of the most likely model was:

$$K_{d}=\;0.037\cdot C_{quartz}\Phi_{n(p<0.001)}+\;0.174\cdot C_{oc}\Phi_{an(p<0.001)}\;R^{2}=0.927$$ (7)

The values of the semipartial correlation of *K*_*d*_ with *C*_*quartz*_ and *K*_*d*_ with *C*_*oc*_ were 0.412 and 0.312, respectively. These values indicate that in the examined pH range quartz had a greater affinity for the neutral form of bentazone than the affinity of organic matter for bentazone anions.

The *R*^*2*^ value of 0.382 was obtained for the model assuming that organic matter was responsible for the adsorption of both bentazone forms, while a semipartial correlation value of 0.122 was obtained for the adsorption of the neutral form and 0.027 only for the adsorption of bentazone anions. From these values, it is understood that quartz had over a three folds greater affinity for the neutral form of bentazone than organic matter. The differences in the prediction of *K*_*d*_ can be clearly identified in Fig 4A. The model assuming adsorption only on organic matter could not predict adsorption in the fractions > 63 μm, in which the content of *C*_*oc*_ was very low (0.01–0.05%). The sum of the predicted by the model six *K*_*d*_ values was underestimated by 29% in comparison to the sum of the experimental values.

According to data presented in Fig 4B, it is noteworthy that, neither lepidocrocite (goethite was not detected in fractions of AR611C) nor the Al and Fe oxides and hydroxides extracted using the Tamm’s solution were significantly involved in adsorption. Thus, adsorption of bentazone on Al and Fe oxides is probably limited to soil groups with properties similar to the Oxisols examined by Bonfleur et al. [7].

The modeling results presented in Fig 4B suggested that quartz is the principal adsorbent of bentazone in acidic sandy soils with a low content of organic matter. However, the experiments were carried out with the fractions of only one soil. To confirm that adsorption really occur on quartz in acidic soils, the mineralogical composition of all the soils from S1 Table in S1 Appendix with pH < 5.0 in 0.01 M CaCl_2_ and *C*_*oc*_ < 0.35% was determined (S5 Table in S3 Appendix). Eq (1) that had the same assumptions as for the AR611C fractions was fitted to the experimental *K*_*d*_ values of the soils (data presented in Fig 1 and in S7 Table in S4 Appendix). The results (Fig 4D) confirmed that quartz was the primary soil mineral responsible for the adsorption of the neutral form of bentazone. The obtained model was as follows:

$$K_{d}=\;0.021\cdot C_{quartz}\cdot \Phi_{n(p<0.001)}+\;0.071\cdot C_{oc}\cdot \Phi_{an(p<0.001)}\;R^{2}=0.911$$ (8)

The value of the semipartial correlation between *K*_*d*_ and *C*_*quartz*_ was 0.643 and between *K*_*d*_ and *C*_*oc*_ was 0.276. However, slightly better results were obtained when the *C*_*quartz*_ variable was replaced with *C*_*sand*_, that is, when the following model was used:

$$K_{d}=\;0.017\cdot C_{sand}\cdot \Phi_{n(p<0.001)}+\;0.078\cdot C_{oc}\cdot \Phi_{an(p<0.001)}\;R^{2}=0.965$$ (9)

**Fig 5:**
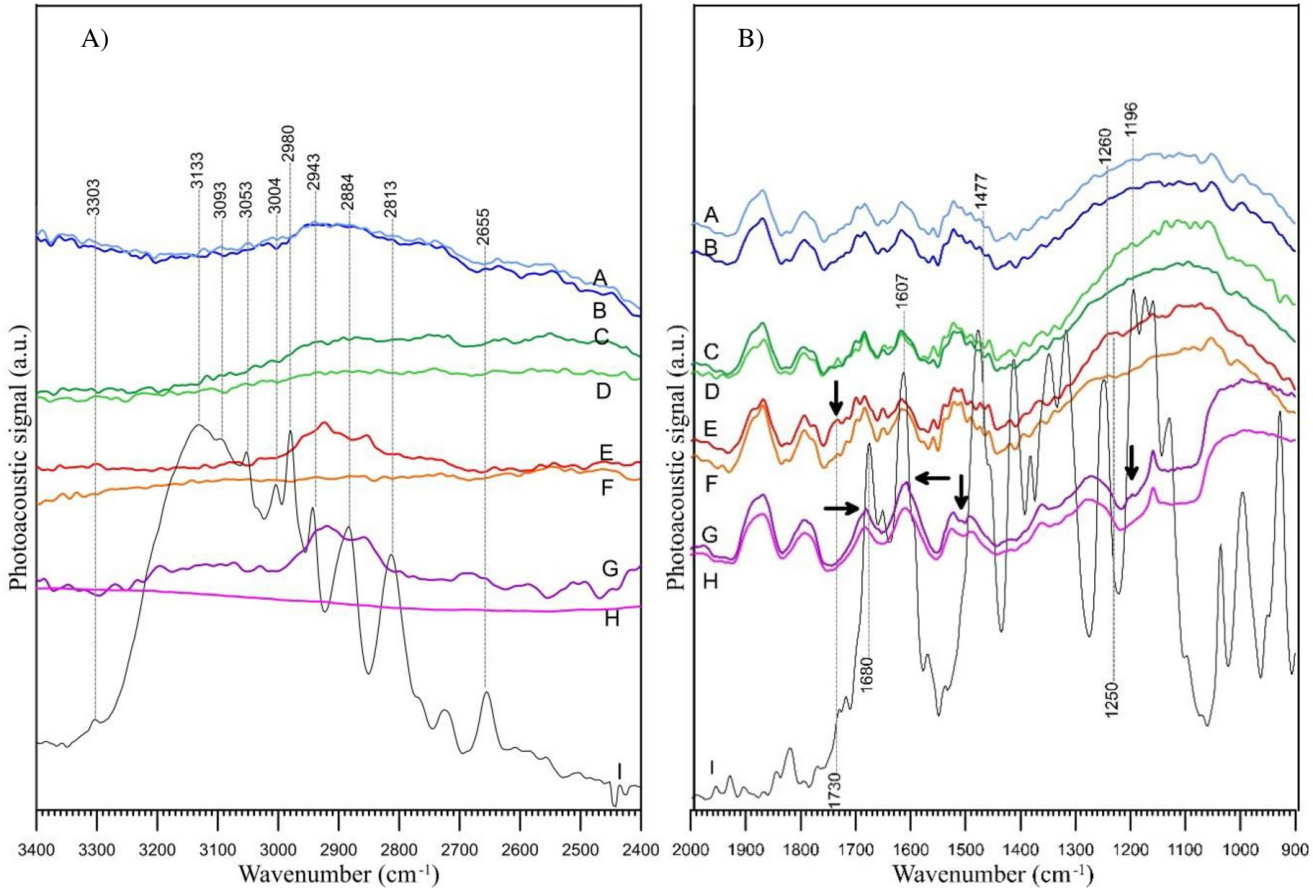
FT-IR/PAS spectra of examined samples of AR774C soil fraction < 500 μm: (A) within 3400–2400 cm^–1^, (B) within 2000–900 cm^–1^. The baselines are displaced vertically to avoid overlaping. The used designations denote: A–soil at pH 7.5, B–soil + bentazone at pH 7.5, C–soil at pH 5.2, D–soil + bentazone at pH 5.2, E–soil at pH 3.5, F–soil + bentazone at pH 3.5, G–soil at pH 1.6, H–soil + bentazone at pH 1.6, and I–pure bentazone.

It was found that the relationship between *C*_*quartz*_ and *C*_*sand*_ was linear, with a high value of Pearson’s correlation coefficient (*r* = 0.949, *p* < 0.001) and lower Kendall’s rank correlation (*r*_*K*_ = 0.576, *p* = 0.055). The second significant positive correlation was found between *C*_*sand*_ and *C*_*albite*_ (*r* = 0.584, *p* = 0.046), whereas the other positive correlations with *C*_*sand*_ were insignificant (S12 Table in S9 Appendix). The results presented in Fig 5A indicate that not all the soil quartz is responsible for the adsorption of bentazone, but first of all the grains of 150–63 μm, and to a much less extent, the grains of 400–150 μm diameter. Therefore, it is not surprising that better results were obtained for Eq (9). As can be seen in S12 Table in S9 Appendix, the value of Kendall’s *r*_*K*_ determined for the nonlinear relationship between *K*_*d*_ and *C*_*sand*_ was 0.546 (*p* = 0.014), while it was only 0.424 (*p* = 0.055) for the relationship between *K*_*d*_ and *C*_*quartz*_.

Fig 4C shows the results for fitting the model assuming the adsorption of both bentazone forms on organic matter. In comparison to the models including the *C*_*quartz*_ or *C*_*sand*_ variables, the results were much worse, which clearly indicates that this assumption is not valid in acidic soils with *C*_*oc*_ < 0.35%. The mean of the predicted by this model *K*_*d*_ values for the 12 soils was underestimated by 22% in comparison to the mean of the experimental ones. In such soils, the variance of the obtained experimental *K*_*d*_ values cannot be explained well without assuming the adsorption of the neutral bentazone form on quartz or sand.

### FT-IR/PAS analyses

IR analyses were performed for obtaining more information on bentazone–quartz interactions. In the case of a sandy soil, choosing the appropriate IR spectroscopic technique seemed to be challenging due to the size and hardness of the sand grains. PAS, attenuated total reflectance (ATR), and diffuse reflectance spectroscopy (DRS) are surface techniques, in which signal is generated from a few micrometers of the surface. These techniques provide information about the chemical structure of the near-surface region. The surface sensitivity for PAS is ~1 μm, while it is ~1 nm for DRS and monolayer for ATR [51]. In the present study, FT-IR/PAS proved to be the best technique, as it provided good–quality spectra when the initial bentazone concentration was increased to 10 mg/L and the fraction of a soil < 500 μm was used. The other two techniques (ATR and DRS) caused problems with sample preparation for IR measurement and the spectra obtained were of poor quality.

The results of FT-IR/PAS are shown in Fig 5. The adsorption of bentazone is confirmed in the spectrum of the AR774C soil fraction < 500 μm at pH 3.5, indicated by the bands at ~1730 and ~1250 cm^–1^ (C = O stretching vibrations) (Fig 5B) and C–H stretching vibrations of methyl groups at ~2960, 2920, and 2850 cm^–1^ (Fig 5A). The C = O stretching absorption is one of the strongest IR absorptions [52], and it is the only band of bentazone that can be seen in the spectrum in Fig 5. The bands corresponding to the aromatic ring or N–H vibration are usually weak in intensity [52]. In pure bentazone the N–H group not involved in hydrogen bond was visible as a weak intensity band at ~ 3303 cm^–1^, whereas the N–H group involved in intermolecular hydrogen bonds was seen as a shifted strong band at 3133 cm^–1^ (Fig 5B). However, the intensities of these bands were too low to be noticed in the spectra obtained after adsorption of the compound. Thus, the only spectral evidence for bentazone adsorption on the soil surface was the presence of the C = O and C–H bands. The C = O bands were shifted from 1680 cm^–1^ in the spectrum of pure bentazone to 1730 cm^–1^ in the spectrum of the AR774C fraction < 500 μm at pH 3.5, and from 1260 to 1250 cm^–1^, respectively. This may indicate that bentazone is entrapped in quartz as a monomer [53] with no involvement of intermolecular hydrogen bonding. Moreover, this shift indicates that bentazone is not bonded to quartz surface via intermolecular hydrogen bonding involving C = O groups. The absence of C = O and C–H bands in the spectra of AR774C fraction <500 μm at pH 5.2 and 7.5 (Fig 5) indicated that adsorption did not occur, or the intensity of the bands was not high enough to be visible in the IR spectra. These observations are consistent with the results presented in Fig 3B.

A slightly different situation was observed for the adsorption of bentazone in the < 500 μm fraction of AR774C at pH 1.6. Some bands were seen at the same wavenumber as observed for the pure compound, while other bands were slightly shifted. In this case, the bands of adsorbed bentazone were visible at ~2943, 2884, and 2861 cm^–1^ (Fig 5A) and at ~1681, 1607 (shifted from 1612 cm^–1^), 1493 (shifted from 1479 cm^–1^), and 1272 cm^–1^ (shifted from 1260 cm^–1^, Fig 5B). The last wavenumber (1260 cm^–1^) may suggest that the quantity of bentazone adsorbed at pH 1.6 was greater than that adsorbed at pH 3.5 (no shift of band toward lower wavenumbers was noticed).

### Mechanisms of bentazone adsorption on the quartz surface

**Fig 6:**
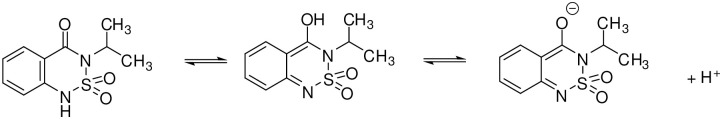
Keto-enol tautomeric equilibrium and dissociation process of bentazone.

The results of adsorption experiments and FT-IR/PAS were supported with computational methods. The literature data show that bentazone may exist in two tautomeric forms [9]. The structure of the compound is of significant importance for the adsorption. To determine the probability of tautomeric transition the total energy for both forms was calculated. To obtain evident results three different approaches (semi empirical PM3, *ab initio* Hartree–Fock model, and density functional (FD) at the selected levels) were used. The data obtained for all models showed that the ketone form is thermodynamically preferred in vacuum as well as in water (S13 Table in S10 Appendix). Thus, the results were consistent with those obtained from the FT-IR spectroscopy. Taking into account the level of energetic barrier for the tautomeric transition and solvation process under the environmental conditions, the equilibrium with the coexisting enol form cannot be excluded (Fig 6). High solvation energy of enol form can promote the transformation. An increase in pH of solution shifts the equilibrium to the right and increases the amount of deprotonated bentazone (Fig 6).

The molecular potential density distribution of the ketone form exhibits that the most negative potential is located on oxygen atoms (= C = O and = SO_2_) and a positive one on amine hydrogen (S9 Fig in S10 Appendix). A charge distribution of the molecule also confirms this finding (S10 Fig in S10 Appendix). This demonstrates the possibility of hydrogen bonds formation by both of = C = O and = N–H groups of the ketone form of bentazone.

The explanation of pH-dependent adsorption mechanisms requires investigations of pH-dependent changes in the adsorbate and adsorbent surface structures. In the case of quartz, the pH-dependent composition of its surface in the aqueous solutions of pH 0–10 was well explained by Duval et al. [48]. According to the 2-pK surface capacitance model with the equilibrium constants (pK_1_ = -1, and pK_2_ = 4) derived experimentally from X-ray photoelectron spectroscopy, the concentration of the ≡ SiOH_2_^+^ species was the highest on the quartz surface at pH 0. As pH increased the ≡ SiOH_2_^+^ groups were deprotonated. The highest concentration of the surface ≡ SiOH groups was observed in the pH range of 2–6, with the maximum one around pH 4. The amount of the ≡ SiO^-^ groups increased from its minimum at pH 0, at pH > 2.2 their amount exceeded that of ≡ SiOH_2_^+^ groups, and at pH > 9 exceeded the amount of ≡ SiOH groups. Similar protonation reactions and surface species occur also on the silica surface [54].

**Fig 7:**
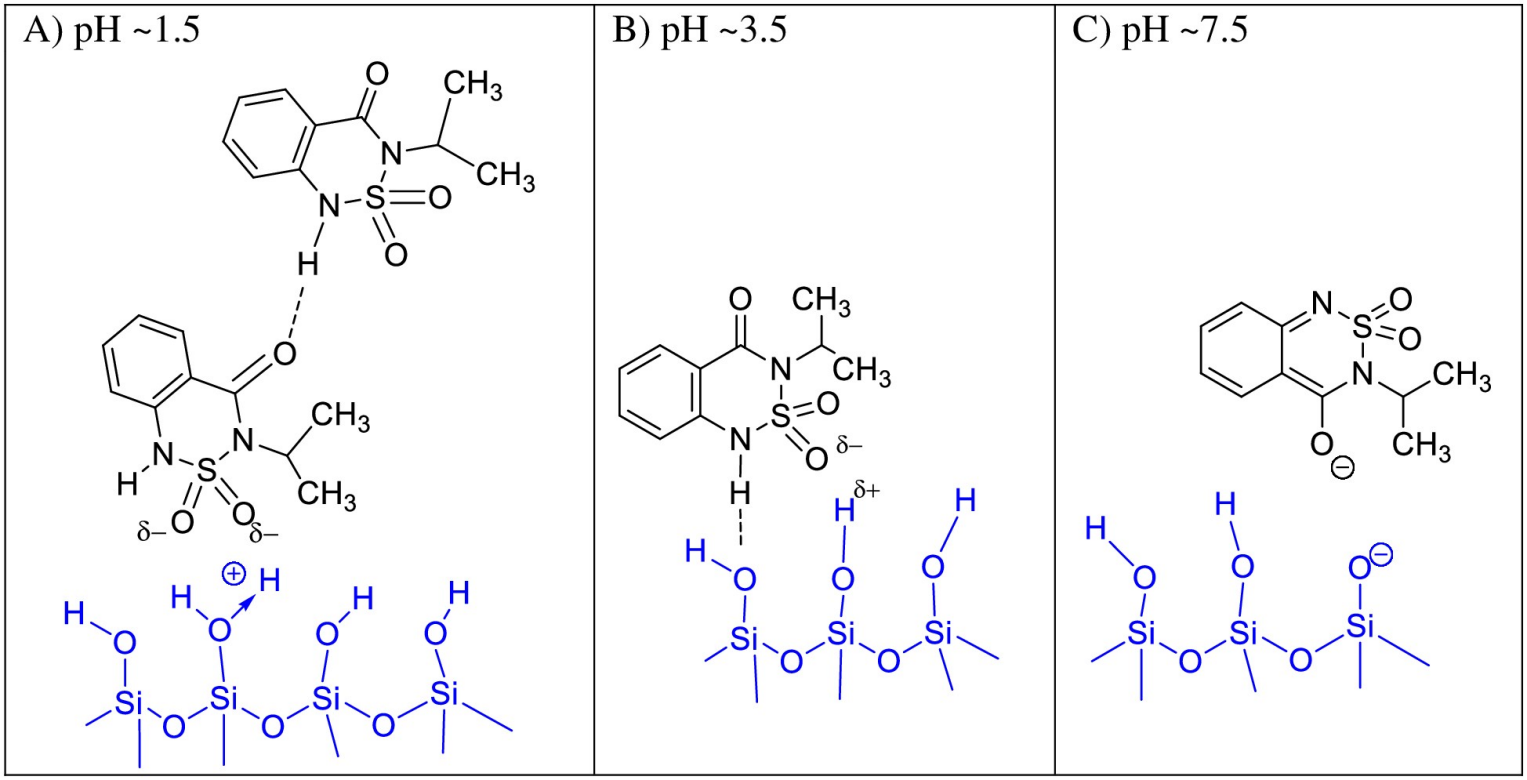
pH-dependent mechanisms of bentazone adsorption on the silanol groups of quartz.

At pH in the range of 3.5–5.0 (Fig 3B) the ketone form of bentazone is adsorbed through the hydrogen bonds involving = N–H groups as hydrogen atom donors (Fig 7B). This is consistent with the results of FT-IR spectroscopy obtained at pH 3.5 (Fig 5). This classic hydrogen bond can be supported by a weaker one, with oxygen atoms of = SO_2_ group. A large negative charge on an oxygen atom justifies this type of interactions, or at least electrostatic interactions (S10B Fig in S10 Appendix).

At pH above the point of zero charge, quartz surface is negatively charged. The number of the negative sites increases with the increasing pH. However, strong deprotonation of the silanol surface sites starts at circumneutral pH [48,55]. The number of bentazone anions also increases along with pH. As it was mentioned earlier, at pH > 5 bentazone exists almost exclusively in the deprotonated anionic form. Therefore, adsorption in the pH range of 3.5–5.0 decreased, and at pH > 5 was not observed (compare Figs 3B, 5, 7B and 7C).

At pH ~1.5 the surface of the silica or quartz is positively charged (Fig 7A). According to Duval et al. [48] and Lowe et al. [54] the Si-OH_2_^+^ groups are present at extremely low pH (~ 2 or lower). In this case, the mechanism of adsorption based on electrostatic interactions seems to be most likely. Taking into account the distribution of charge, the most negative areas of the molecule are those with = SO_2_ oxygen atoms (S9 and S10 Figs in S10 Appendix). Bentazone molecules are adsorbed probably as dimers. The FT-IR studies, showed that the = C = O group is involved in the hydrogen bond (Fig 5). However, dimers can orientate differently on the surface (only one molecule in the pair or both can interact with the surface), which can determine the range of adsorption (Fig 7A).

Summarizing, according to the proposed model, depending on the pH, either hydrogen-bonding or ionic (ion-dipol) interactions are the significant mechanisms of bentazone adsorption on quartz.

## Conclusions

Bentazone was very weakly adsorbed in the examined mineral soils, especially in subsoils that had a low content of organic matter. The results of the PLSR analysis indicated that both the neutral and anionic forms of bentazone were adsorbed on organic matter, while the neutral form was also adsorbed on sand.

The adsorption experiments conducted on soils with no or low content of organic matter and the detailed analyses of the mineralogical composition of the soils, indicated that in such soils bentazone molecules were adsorbed on quartz surface. In soils with a pH < 5 and an organic carbon content of < 0.35%, quartz was found to have much greater affinity for the neutral bentazone form of than organic matter. The quartz content in soils was strongly correlated with their sand content. Adsorption experiments conducted on individual soil fractions indicated that the strongest adsorption on quartz occurred in the fraction of 150–63 μm. The created based on nonlinear weighted regression model, assuming the adsorption of bentazone on soil organic matter and on sand and using the spectrophotometrically determined *pK*_*a*_ of bentazone, very well explained the *K*_*d*_ variance in 81 examined soils, and correctly predicted *K*_*d*_ values based on soil properties described in the published data.

The proposed model should be applicable primarily for soils of the temperate climate zone, in which the content of Fe and Al oxides and hydroxides is low. The presence of adsorption on quartz means that bentazone can be bound in the sand fractions of soil profiles, in sandy formations being in contact with groundwater contaminated with bentazone, and in sandy sediments of contaminated surface waters. At pH < 5 adsorption in these formations/sediments should be significantly higher than it would appear from their organic matter content. In the case of bentazone, this phenomenon has so far been overlooked.

Molecular modeling studies have shown that bentazone mainly occurs in the ketone form. FT-IR/PAS studies combined with computational methods have explained the mechanism behind the adsorption of bentazone on quartz. At pH < 5 bentazone was shown to bind to quartz surface probably through the formation of hydrogen bonds. At pH around 1.5, the electrostatic interactions between the positively charged surface of quartz and the areas of the molecule with accumulated negative charges seemed to be decisive. At pH > 5 bentazone occurs in the anionic form and no adsorption on quartz surface was recorded.

## Supporting information

S1 Appendix Properties of soils used for adsorption experiments. **S1 Table.** Basic physical and chemical properties and locations of soils from 27 profiles of AR, LV and LV&CM soil groups. **S1 Fig.** Locations of 27 soil profiles chosen for the study on the map of Poland.**S2 Fig.** Photographs from the binocular magnifier (left side) and the polarizing optical microscope (thin sections, crossed polars, right side) of 12 selected Arenosols and Luvisols.
(PDF)

S2 Appendix Determination the *pKa* of bentazone. **S2 Table.** Volumes of HCl, KCl, KOH and H_2_O used for the preparation of solutions used to stabilize pH and ionic strength during measurements the bentazone absorbance. **S3 Table.** Absorbance and pH of solutions measured to determine the *pK*_*a*_ of bentazone. **S4 Table.** Results of fitting Eq (S1) to data presented in S2 Table.
(PDF)

S3 Appendix Sample preparation and X-ray powder diffraction. **S5 Table.** Mineralogical composition (%) of the 12 soils selected from S1 Table with pH in 0.01 M CaCl_2_ < 5.0 and *C*_*oc*_ < 0.35%. **S3 Fig.** Sample diffractograms of soil samples from 76 (NW Poland), 611 (CE Poland) and 872 (SE Poland) profiles. Acronyms denote: bt–biotite, cl–clinochlore, epi–epidote, gl–glauconite, il–illite, ka–kaolinite, mu–muscovite, or–orthoclase, qzt–quartz, ref–CaF_2_, and ru–rutile.
(PDF)

S4 Appendix Adsorption kinetics and *K*_*d*_ values from batch experiments. **S4 Fig.** The results of the batch kinetic experiments. **S6 Table.** The results of fitting Eq (S5) to the batch experiment data. **S7 Table.** *K*_*d*_ values obtained from batch experiments for soils from S1 Table.
(PDF)

S5 Appendix Correlations between soil properties and *K*_*d*_. **S8 Table.** Kendall rank correlation matrix for soil properties and *K*_*d*_ (n = 81 soils).
(PDF)

S6 Appendix Comparison of *K*_*d*_ values available in literature. **S9 Table.** *K*_*d*_ values for bentazone available in literature.
(PDF)

S7 Appendix Determination of the point of zero net proton charge. **S5 Fig.** The point of zero net proton charge (PZNPC) of the < 500 μm AR774C fraction.
(PDF)

S8 Appendix Adsorption in fractions of AR611C. **S6 Fig.** Fractions of AR611C –photographs from the binocular magnifier showing quartz, orthoclase, chalcedonite, glauconite and accessory minerals. **S7 Fig.** Backscattered scanning electron (*BSE*) micrographs of the AR611C grains: fraction 150–63 μm (a), typical quartz grain surface with v-shaped holes and adsorbed clay minerals (b). **S8 Fig.** Diffractograms of fractions of AR611C. Acronyms denote: bt–biotite, cl–clinochlore, epi–epidote, gl–glauconite, mu–muscovite, or–orthoclase, qzt–quartz, ref–CaF_2_ and ru–rutile. **S10 Table.** Results of adsorption experiments using the fractions obtained by sieving the native AR611C soil. **S11 Table.** Properties of AR611C fractions used for the adsorption experiments.
(PDF)

S9 Appendix Adsorption in 12 selected soils with pH < 5.0 and *C*_*oc*_ < 0.35%. **S12 Table.** Kendall (bottom, left) and Pearson (top, right) correlation coefficients (n = 12).
(PDF)

S10 Appendix Modeling of bentazone adsorption on quartz surface. **S9 Fig.** Molecular electrostatic potentials (MEPs) of bentazone. The deepest blue color denotes the most positive potential, the deepest red color–the most negative potential, and intermediate shades–the intermediate potential regions (calculated at RHF 6–311+G** basis set). **S10 Fig.** The charge distribution of the ketone (a, b) and enol (c, d) tautomers of bentazone: Mullicen charge (a, c), electrostatic charge (b, d). Calculations at RHF 6–311+G** basis set. **S13 Table.** Total energy (E_T_ (au)) of bentazone tautomers calculated on different ways.
(PDF)
