# Supplementary material for: Adsorption of bentazone in the profiles of mineral soils with low organic matter content
Source: PLoS One. 2020 Dec 2;15(12):e0242980. doi: 10.1371/journal.pone.0242980 (PMC7710104; doi:10.1371/journal.pone.0242980)
Supplement: S1 Appendix — S1 Table. Basic physical and chemical properties and locations of soils from 27 profiles of AR, LV and LV&CM soil groups. S1 Fig. Locations of 27 soil profiles chosen for the study on the map of Poland.S2 Fig. Photographs from the binocular magnifier (left side) and the polarizing optical microscope (thin sections, crossed polars, right side) of 12 selected Arenosols and Luvisols. (PDF) [file pone.0242980.s001.pdf]

A Appendix. Properties of soils used for adsorption experiments.

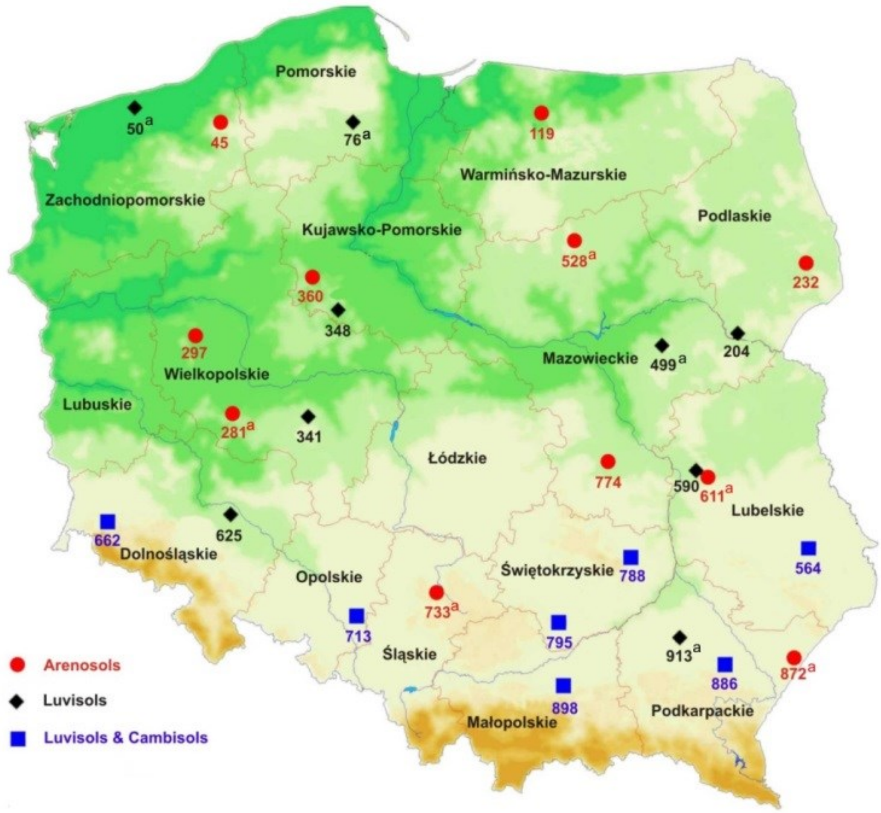

[a] soil profiles from which 12 soils with pH in 0.01 M $CaCl_2$ < 5.0 and C_{oc} < 0.35% (S1 Table) were selected for the detailed studies on their mineralogical composition.

S1 Fig. Locations of 27 soil profiles chosen for the study on the map of Poland.

S1 Table. Basic physical and chemical properties and locations of soils from 27 profiles of AR, LV and LV&CM soil groups.

Prof. No.	Location[a]	Hor.	Depth (cm)	C_{sand}[b] (%) 2000-50 µm	$C_{cos+vcos}$[c] (%) 2000-500 µm	C_{ms}[c] (%) 500-250 µm	C_{fs+vfs}[c] (%) 250-50 µm	C_{silt}[b] (%) 50-2 µm	C_{cosi}[c] (%) 50-20 µm	C_{fsi}[c] (%) 20-2 µm	C_{clay}[b] (%) < 2 µm	C_{oc}[d] (%)	C_{Al}[e] (mg/kg)	C_{Fe}[e] (mg/kg)	C_{Mn}[e] (mg/kg)	pH ($CaCl_2$)
							**Arenosols (AR)**									
45	Gołdaw	Ap	10-15	82.2	2.4	21.2	58.5	15.4	6.3	9.1	2.4	1.13	22.50	0.98	29.4	5.0
	(53˚59´N, 16˚34´E)	BC	35-40	93.9	11.0	30.8	52.1	4.8	2.2	2.5	1.3	0.10	0.79	0.67	1.6	5.8
		C	90-95	93.5	15.7	34.2	43.6	4.8	2.0	2.8	1.7	0.02	0.97	1.12	1.2	6.0
119	Wróblik	Ap	20-25	90.2	32.7	35.1	22.4	7.0	2.3	4.6	2.8	1.42	45.13	1.45	83.8	4.6
	(54˚04´N, 20˚27´E)	Bw	60-65	96.5	2.9	34.3	59.3	1.9	1.2	0.7	1.6	0.33	9.94	0.67	2.3	5.5
		C	100-105	97.5	4.3	37.4	55.8	1.0	0.5	0.4	1.5	0.06	4.47	0.60	2.8	5.5
232	Makówka	Ap	10-20	92.7	0.5	21.0	71.2	5.4	1.6	3.9	1.9	0.88	46.85	2.02	34.5	4.2
	(52˚54´N, 23˚33´E)	Bw	35-45	98.1	0.0	14.5	83.6	1.1	0.5	0.6	0.8	0.35	22.20	0.82	4.6	5.1
		C	90-100	98.7	0.0	23.0	75.7	0.4	0.2	0.2	0.9	0.00	0.98	0.52	2.7	5.5
281	Górzno	Ap	10-20	87.8	4.5	26.5	56.8	9.1	3.9	5.2	3.1	1.06	62.67	4.92	9.9	3.9
	(51˚53´N, 16˚49´E)	Bw	40-50	94.5	3.9	28.0	62.6	4.7	2.4	2.3	0.8	0.31	25.14	2.87	3.3	4.8
		C	90-100	95.4	20.5	40.1	34.9	2.6	1.1	1.5	2.0	0.07	5.76	0.60	2.2	5.2
297	Śliwno	Ap	0-30	83.9	5.1	19.0	59.7	14.3	6.5	7.8	1.8	1.09	36.33	2.03	29.8	4.7
	(52˚26´N, 16˚22´E)	BC	30-50	88.2	8.7	29.1	50.4	9.6	5.9	3.7	2.2	0.10	4.04	0.68	5.4	5.6
		C	80-100	94.9	16.0	41.9	37.1	3.2	1.5	1.7	1.9	0.05	0.66	0.38	11.4	5.8
360	Dobrylewo	Ap	10-20	92.1	0.2	12.5	79.4	5.1	1.6	3.5	2.8	0.69	4.40	0.82	59.2	5.2
	(52˚53´N, 17˚43´E)	BC	40-50	96.9	0.0	9.7	87.2	1.6	0.4	1.2	1.5	0.08	0.34	0.75	36.1	6.4
		C	100-110	97.9	0.0	7.0	90.9	0.3	0.1	0.2	1.8	0.00	0.20	0.60	4.7	6.6
528	Marianowo	Ap	10-25	84.4	6.1	31.3	47.0	13.8	5.9	7.8	1.8	1.25	5.34	0.52	16.5	5.4
	(53˚08´N, 20˚49´E)	BC	40-60	94.1	7.1	34.9	52.2	3.8	2.1	1.7	2.1	0.09	32.63	0.45	5.3	4.8
		C	70-90	97.5	4.3	45.5	47.8	0.7	0.4	0.3	1.8	0.05	4.42	0.37	1.7	5.4
611	Olempin	Ap	5-10	87.5	11.9	32.6	43.1	10.1	5.5	4.6	2.4	0.68	35.20	1.80	11.3	4.1

	(51°24´N, 22°14´E)	BC	35-45	92.6	13.4	35.5	43.8	5.7	2.7	3.1	1.7	0.09	25.38	0.99	1.9	4.6
		C	65-75	96.1	0.3	21.5	74.3	2.4	1.0	1.4	1.5	0.03	24.50	0.90	4.0	4.3
733	Rosochacz	Ap	0-25	83.3	2.3	24.4	56.7	14.8	7.0	7.8	1.9	1.30	33.57	3.37	11.3	4.9
	(50°37´N, 19°12´E)	Bw	25-60	92.0	2.3	30.8	58.9	5.8	3.4	2.4	2.2	0.48	60.03	1.57	10.6	4.6
		C	60-150	94.3	3.5	35.4	55.5	3.8	2.1	1.7	1.9	0.06	27.68	0.89	5.3	4.5
774	Jedlińsk	Ap	10-20	89.3	1.1	17.1	71.1	8.7	3.5	5.2	2.0	1.11	26.41	2.09	26.5	4.4
	(51°32´N, 21°08´E)	Bw	40-50	95.1	4.9	25.0	65.2	3.9	2.1	1.7	1.0	0.30	16.89	1.05	8.0	5.1
		C	105-110	96.9	0.0	6.7	90.2	1.0	0.1	0.8	2.1	0.00	0.21	0.82	0.0	6.1
872	Łukawiec	Ap	10-20	92.1	1.0	25.0	66.0	6.1	2.2	4.0	1.8	0.80	60.55	1.05	31.9	4.2
	(50°04´N, 23°08´E)	Bw	60-70	96.3	0.8	28.9	66.6	2.0	0.7	1.3	1.7	0.17	24.93	1.19	23.1	4.7
		C	100-110	98.0	1.4	28.9	67.7	0.8	0.3	0.4	1.2	0.03	39.27	1.57	9.9	4.4
							**Luvisols (LV)**									
50	Niemierze	Ap	5-10	71.5	2.4	15.7	53.5	21.8	7.2	14.6	6.7	1.23	6.21	1.65	39.3	5.2
	(54°04´N, 15°31´E)	E	30-35	71.2	1.4	14.0	55.7	21.5	8.7	12.7	7.3	0.35	35.99	0.60	52.6	4.5
		Bt	90-95	61.0	2.7	12.4	45.9	21.5	10.0	11.5	17.5	0.15	4.17	0.90	11.3	5.3
76	Wilcze Błota	Ap	10-30	73.3	3.4	15.6	54.3	18.6	6.6	12.1	8.1	1.23	2.73	1.05	38.7	5.2
	(54°01´N, 18°10´E)	Bt1	40-60	55.4	0.5	8.9	46.1	22.9	11.6	11.3	21.7	0.18	59.51	1.41	4.5	4.6
		Bt2	80-100	59.3	1.0	8.6	49.7	18.6	10.0	8.6	22.1	0.11	76.85	0.60	2.7	4.5
204	Drohiczyn	Ap	5-15	71.6	1.1	14.9	55.7	20.9	10.3	10.6	7.5	1.69	0.60	1.12	57.0	6.1
	(52°25´N, 22°41´E)	E	30-40	76.4	0.8	14.5	61.1	17.9	7.1	10.8	5.7	0.36	0.07	1.05	10.0	6.3
		Bt	100-110	49.7	0.2	5.9	43.6	25.5	10.9	14.6	24.8	0.08	0.00	1.20	6.6	6.7
341	Fabianów	Ap	10-20	68.9	0.8	15.0	53.0	21.7	8.0	13.6	9.4	1.16	0.11	0.89	23.8	6.8
	(51°53´N, 17°41´E)	Bt1	40-45	67.7	1.4	12.8	53.5	18.1	9.2	8.9	14.2	0.26	0.05	0.60	5.3	6.5
		Bt2	100-110	56.4	1.1	11.6	43.8	21.9	9.8	12.1	21.7	0.13	0.25	0.91	0.5	6.0
348	Olsza	Ap	10-30	86.3	19.0	27.7	39.6	9.8	3.4	6.4	3.9	0.72	0.48	1.49	134.6	5.7
	(52°39´N, 18°01´E)	E	30-50	78.2	0.3	12.7	65.3	16.5	7.0	9.6	5.3	0.13	0.03	0.38	9.3	6.4
		Bt	80-100	60.2	0.4	8.1	51.7	21.3	11.0	10.3	18.5	0.09	0.01	0.38	6.0	6.3
499	Pniewnik	Ap	0-25	80.7	1.3	17.1	62.3	14.4	5.9	8.6	4.9	1.29	57.96	5.55	62.0	4.2

	(52°22´N, 21°48´E)	E	25-45	72.4	1.2	15.8	55.4	18.8	7.6	11.2	8.8	0.20	48.33	0.45	39.1	4.5
		Bt	45-110	59.1	1.2	13.0	44.9	23.8	10.3	13.5	17.1	0.16	21.59	1.04	21.9	4.9
590	Dęba	Ap	5-15	80.0	4.5	17.2	58.3	16.5	8.2	8.3	3.5	0.93	25.57	1.05	32.7	4.7
	(51°26´N, 22°10´E)	E	35-45	79.8	3.7	16.8	59.2	15.3	7.2	8.1	4.9	0.12	6.22	1.06	14.8	5.0
		Bt	65-75	60.0	1.6	9.8	48.7	20.6	10.7	9.9	19.4	0.08	19.63	0.67	9.9	4.9
625	Krępice	Ap	10-20	69.1	2.4	15.7	51.0	22.6	7.9	14.8	8.3	1.17	3.81	1.35	78.1	5.3
	(51°10´N, 16°49´E)	Bt	50-55	42.0	0.9	7.1	34.0	30.0	12.7	17.3	28.0	0.15	0.04	0.30	1.2	6.3
		BC	110-115	49.8	1.7	11.0	37.1	27.6	11.6	15.9	22.6	0.07	6.34	0.74	33.0	5.0
913	Werynia	Ap	5-20	72.0	3.0	20.4	48.6	20.9	8.5	12.4	7.1	0.91	49.79	1.49	122.2	4.4
	(50°15´N, 21°52´E)	Bt	40-50	50.6	1.2	8.4	41.0	23.4	12.4	11.0	26.0	0.15	260.07	1.49	13.9	4.3
		BC	80-90	56.6	2.1	13.0	41.5	23.9	11.7	12.2	19.5	0.06	7.75	0.22	23.3	5.1
							**Luvisols or Cambisols (LV&CM)**									
564	Skierbieszów	Ap	10-20	25.5	0.3	1.0	24.3	64.8	44.6	20.2	9.7	1.23	0.13	0.30	19.6	6.6
	(50°51´N, 23°22´E)	Bw	35-50	18.5	0.0	0.0	18.5	71.7	46.4	25.2	9.8	0.53	0.03	0.45	14.8	6.3
		BC	65-75	21.3	0.0	0.0	21.3	68.3	43.4	24.9	10.4	0.49	0.01	1.65	6.0	6.2
662	Ubocze	Ap	15-25	16.0	0.3	1.8	13.9	80.4	39.7	40.6	3.6	1.80	0.18	0.89	4.0	7.2
	(51°04´N, 15°26´E)	Bw	35-45	21.3	0.4	1.6	19.4	74.5	45.2	29.3	4.2	0.99	2.20	0.15	72.4	5.5
		Bt	100-110	26.0	0.5	3.2	22.3	63.9	38.8	25.1	10.1	0.31	7.05	0.38	2.8	5.3
713	Klucz	Ap	0-35	16.6	0.0	0.0	16.5	81.3	45.2	36.1	2.1	1.33	0.15	0.15	18.7	6.8
	(50°26´N, 18°17´E)	Bt1	35-75	18.4	0.0	0.0	18.4	67.9	41.4	26.5	13.7	0.24	0.00	0.53	8.0	6.2
		Bt2	75-150	14.9	0.0	0.0	14.9	69.2	38.8	30.5	15.9	0.13	24.75	0.53	7.8	5.1
788	Łężyce	Ap	10-20	16.8	0.0	0.0	16.8	73.2	42.9	30.3	10.0	1.05	0.30	0.53	6.4	7.1
	(50°49´N, 21°20´E)	Bw	25-35	21.5	0.0	0.0	21.4	67.3	46.0	21.3	11.2	0.56	0.03	0.91	0.0	7.3
		BC_{ca}	110-120	16.3	0.0	0.0	16.3	77.4	48.1	29.3	6.3	0.46	0.02	0.74	0.0	7.6
795	Złota	Ap	10-20	14.6	0.0	0.0	14.6	78.5	43.7	34.8	6.9	1.02	3.97	0.60	161.8	5.1
	(50°23´N, 20°34´E)	Bw	30-40	22.0	0.0	0.0	22.0	69.1	44.8	24.4	8.9	0.27	0.00	1.65	14.0	6.4
		BC_{ca}	110-120	19.6	0.0	0.0	19.6	73.8	49.2	24.7	6.6	0.47	0.00	0.15	0.7	7.7
886	Studzian	Ap	5-15	15.1	0.0	0.0	15.1	74.6	41.3	33.3	10.3	1.27	3.36	0.53	74.5	5.2

	(50°03′N, 22°22′E)	Bt	50-60	22.7	0.0	0.0	22.7	61.5	41.7	19.9	15.8	0.20	0.01	0.08	5.4	6.2
		BC	110-120	22.6	0.0	0.0	22.6	68.1	48.3	19.8	9.3	0.12	0.09	0.08	7.4	6.6
898	Uszwica Porębska	Ap	15-25	13.0	0.0	0.0	13.0	80.7	44.5	36.2	6.3	1.39	9.47	0.67	103.0	5.1
	(49°56′N, 20°35′E)	Bt	50-60	17.5	0.0	0.0	17.5	62.8	40.5	22.3	19.7	0.24	19.67	0.30	26.8	4.9
		BC	90-100	14.5	0.0	0.0	14.5	71.5	45.8	25.7	14.0	0.13	43.33	0.00	24.7	5.1

[a] profiles selected and soil samples obtained from the database and soil collection of the Institute of Agrophysics of the Polish Academy of Science in Lublin [1]; [b] determined using the pipette method [2]; [c] determined using a Master Sizer 2000 particle size analyser with a Hydro 2000G adapter, results were corrected for the pipette method (for details see Paszko [3]); [d] determined using a Shimadzu TOC-VCSH analyser and a SSM-5000A solid sample module; [e] determined after extraction with 1 M KCl [4]

Removal of Al and Fe oxides and hydroxides from soil.

For some adsorption experiments, samples of AR774C soil in which the oxides and hydroxides of Al and Fe were removed were used. According to the procedure of Mehra and Jackson [5] 2.2 g of samples were placed in 45 mL polypropylene tubes, and 22 mL of 0.3 M sodium citrate and 2.75 mL of sodium bicarbonate solutions were added. The samples were heated to 80°C in a water bath. Then, 0.28 g of sodium dithionite was added, and the suspensions were mixed for 15 min; this step was carried out twice. After second mixing, 5.5 mL of saturated sodium chloride solution and 5.5 mL of acetone were added, and the samples were heated in a water bath. The tubes were centrifuged (15 min, 2790 g, 20°C), and solution was removed. Then, the soil samples were washed four times with the same volume of redistilled water followed by centrifugation. Finally the samples were dried at 35 °C for 48 h and averaged.

The second procedure was extraction of Al and Fe oxides with the Tamm's solution (0.2 M oxalic acid and ammonium oxalate solution adjusted to pH 3.0) [6]. Briefly, duplicate 1 g soil samples (six fractions of the AR611C were used) were agitated for 4 h with 50 mL of the Tamm's solution and centrifuged (15 min, 2790 g, 20°C); this step was repeated four times. Then, each sample was washed four times with 50 mL of redistilled water followed by centrifugation. The collected solutions were used to Fe and Al determination by means of the atomic absorption spectroscopy (data are presented in S11 Table).

a) AR528BC (53˚08´N, 20˚49´E), d.: 40-60 cm

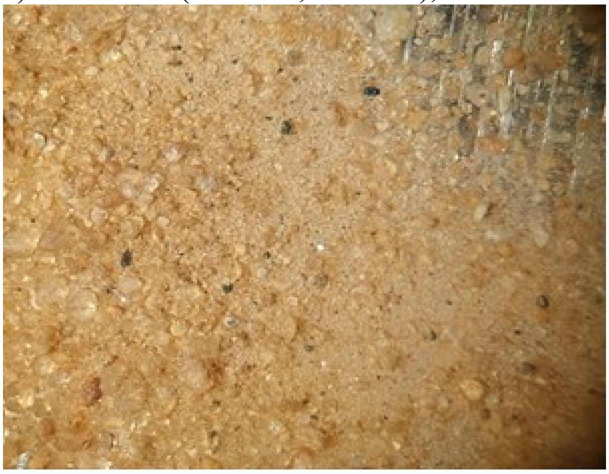

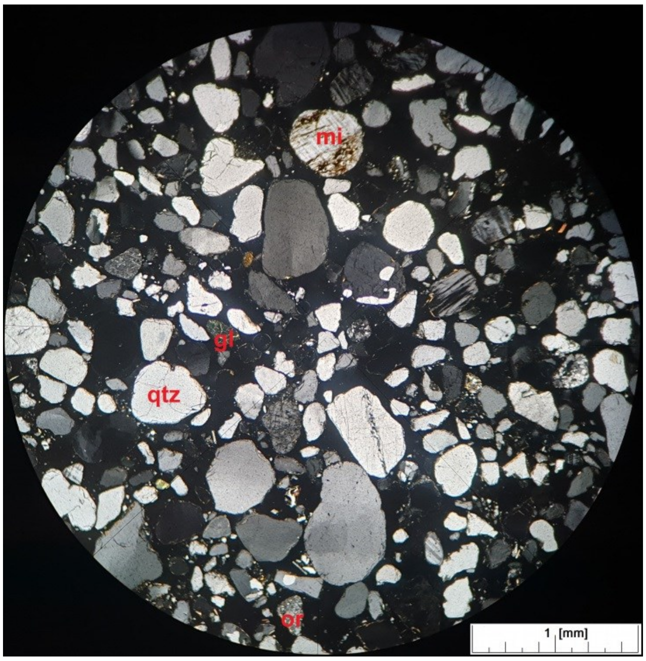

Quartz (qtz), orthoclaze (or) and microcline (mi) crystals as well as glauconite (gl) aggregates.

b) AR281Bw (51˚53´N, 16˚49´E), d.: 40-50 cm

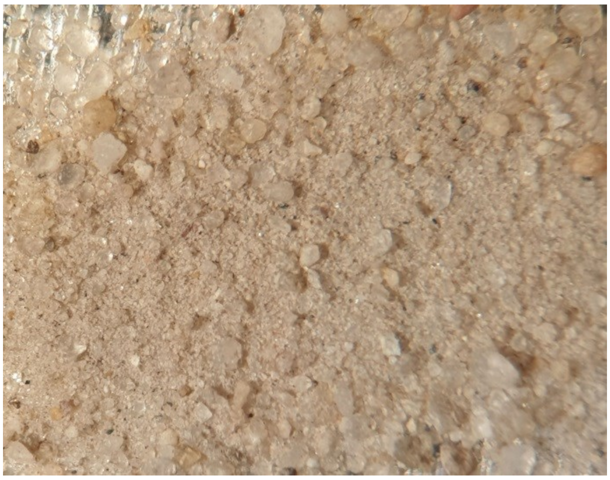

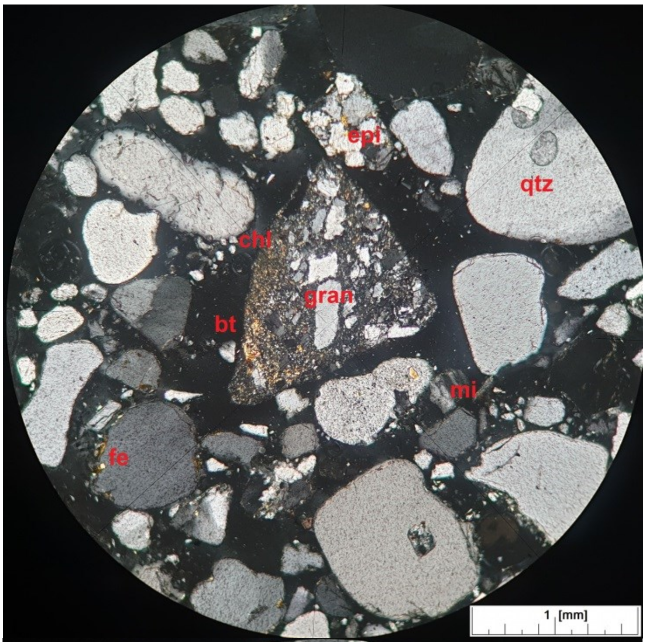

Quartz (qtz), microcline (mi), iron compounds (fe), granite (gran) grain with chlorite (chl), biotite (bt) with quartz.

c) AR872Bw (50˚04´N, 23˚08´E), d.: 60-70 cm

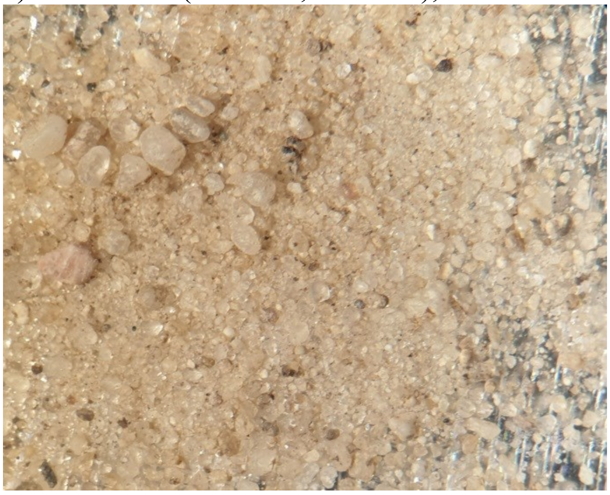

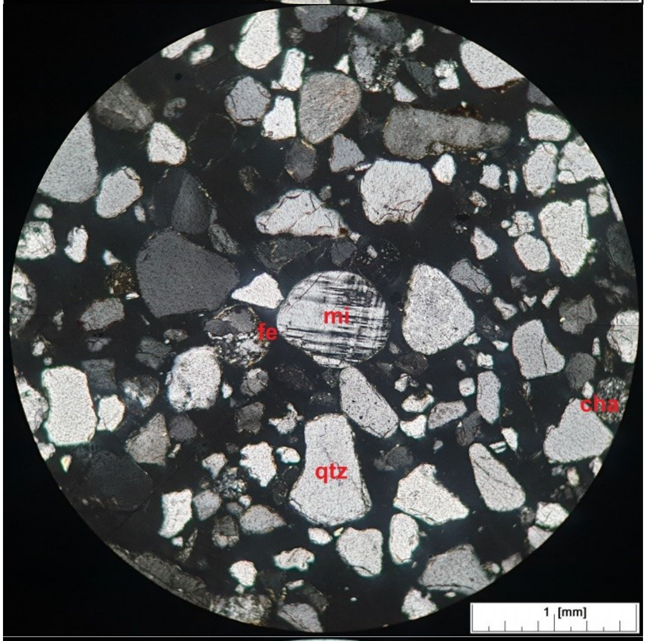

Microcline (mi), quartz (qtz), iron compounds (fe) and chalcedony (cha).

d) AR872C (50˚04´N, 23˚08´E), d.: 100-110 cm

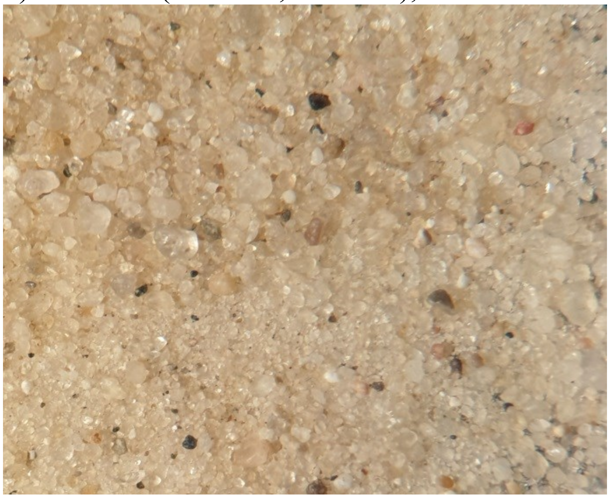

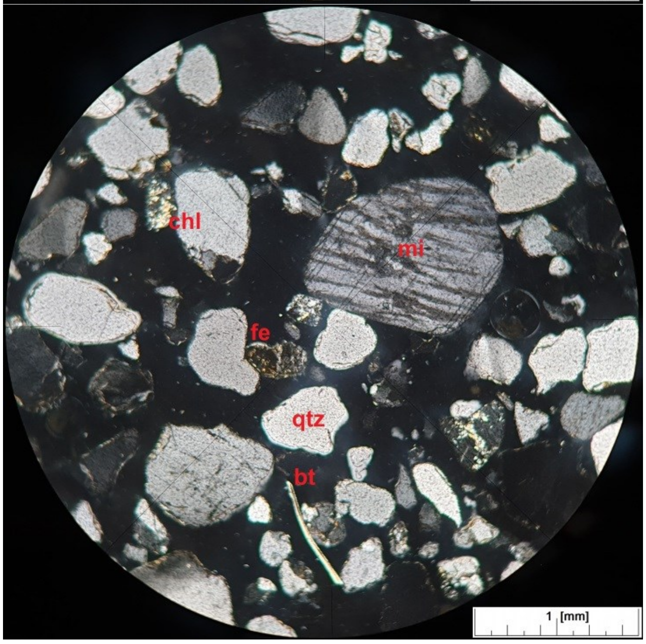

Microcline (mi), iron compounds (fe), quartz (qtz), biotite (bt) and chlorite (chl).

e) AR611BC (51˚24´N, 22˚14´E), d.: 35-45 cm

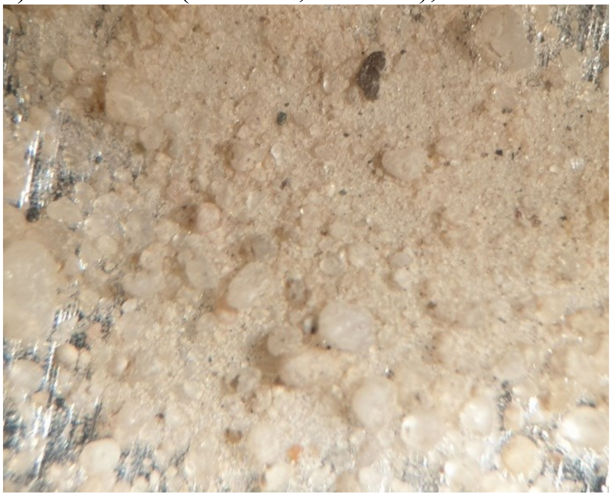

Quartz (qtz), biotite (bt), glauconite (gl) and quartzite (qtzt) with hematite (hem).

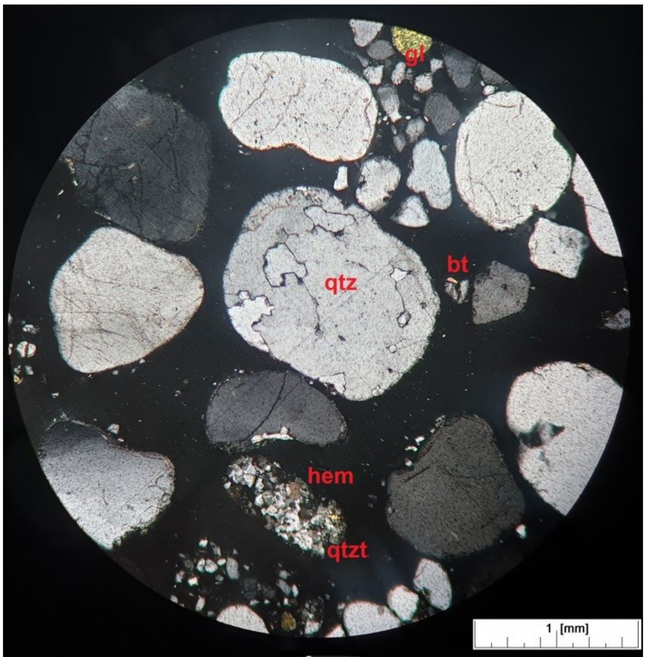

f) AR611C (51˚24´N, 22˚14´E), d.: 65-75 cm

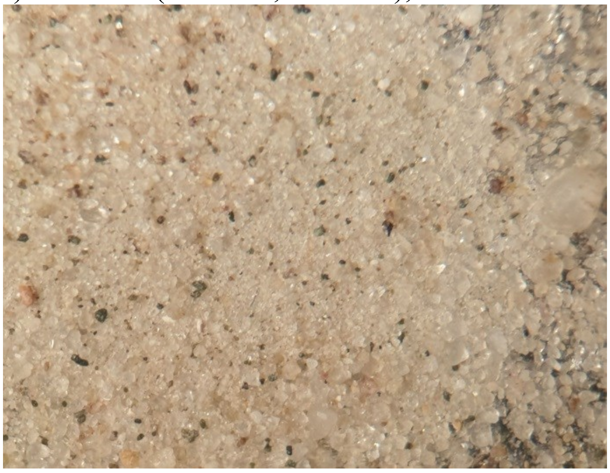

Quartz (qtz), orthoclase (or), quartzite (qtzt) epidote (epi) grains, clay minerals (cl), iron compounds (fe), chlorite (chl) and redeposited glauconite (gl) aggregates.

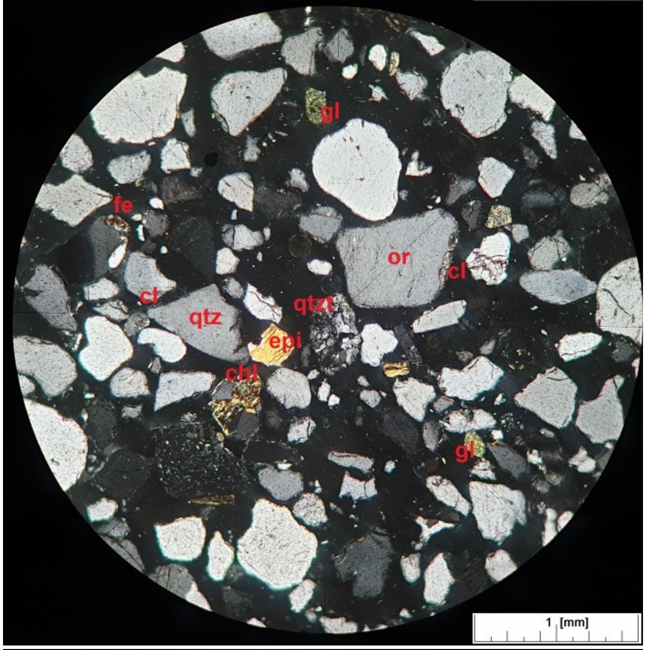

g) LV50E (54˚04´N, 15˚31´E), d.: 30-35 cm

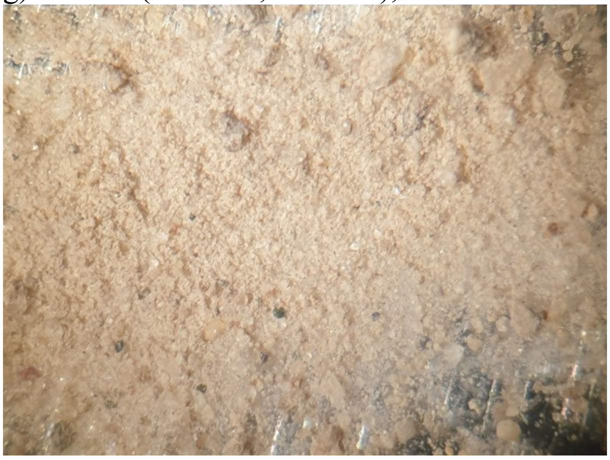

Quartz (qtz) and chalcedonite (cha) grains with biolite (bt), chlorite (chl), sericite (ser), orthoclase (or), clay minerals (cl) and glauconite (gl).

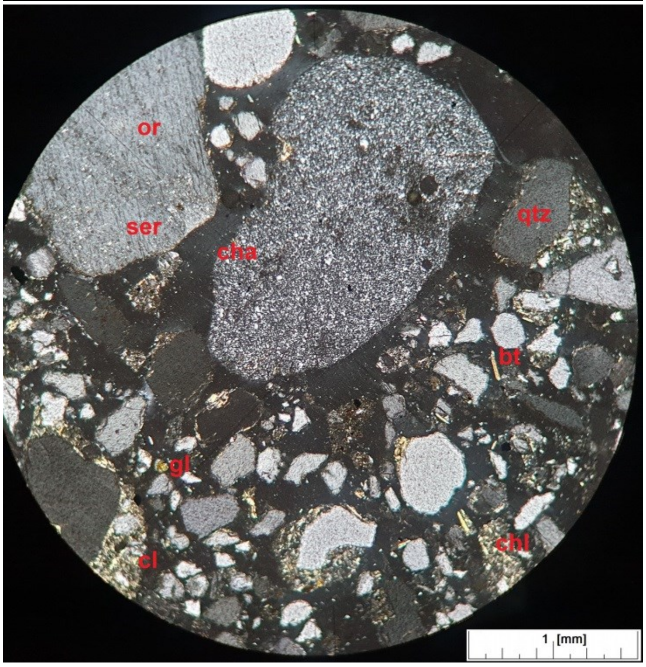

h) LV76Bt1 (54˚01´N, 18˚10´E), d.: 40-60 cm

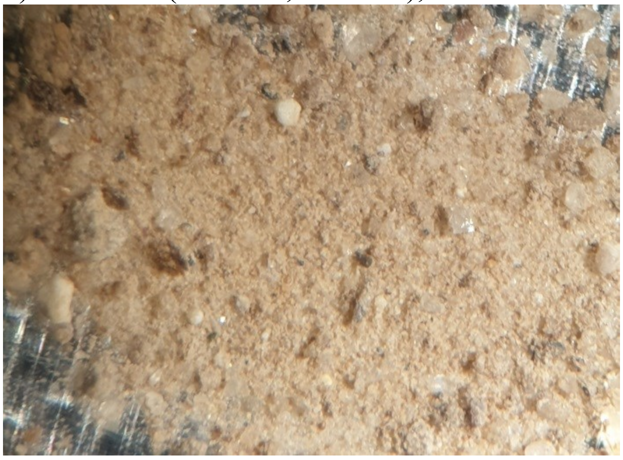

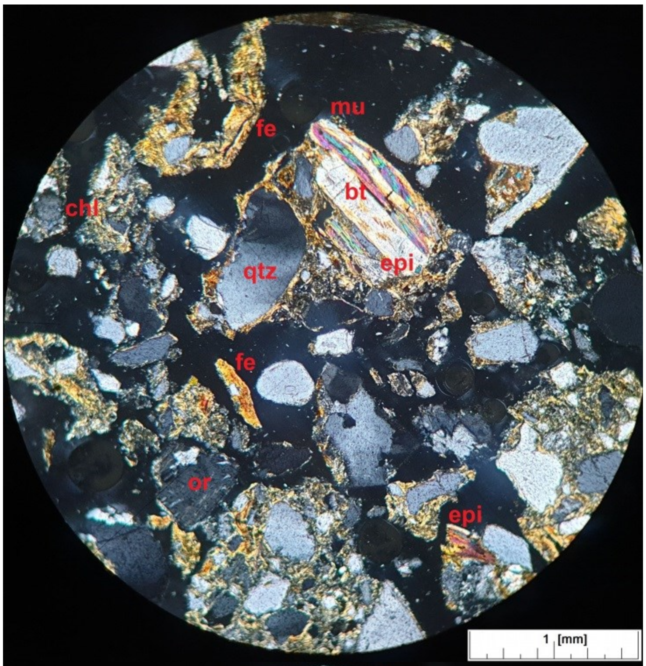

Quartz grains (qtz), orthoclase (or) and gneiss grains with biotite (bt), chlorite (chl), epidote (epi), muscowite (mu) and iron compounds (fe).

i) LV76Bt2 (54˚01´N, 18˚10´E), d.: 80-100 cm

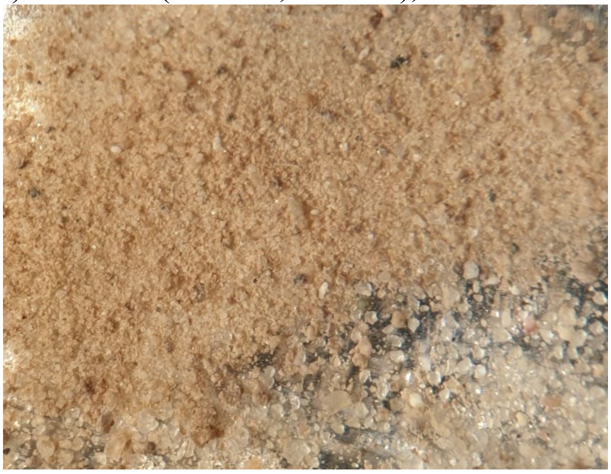

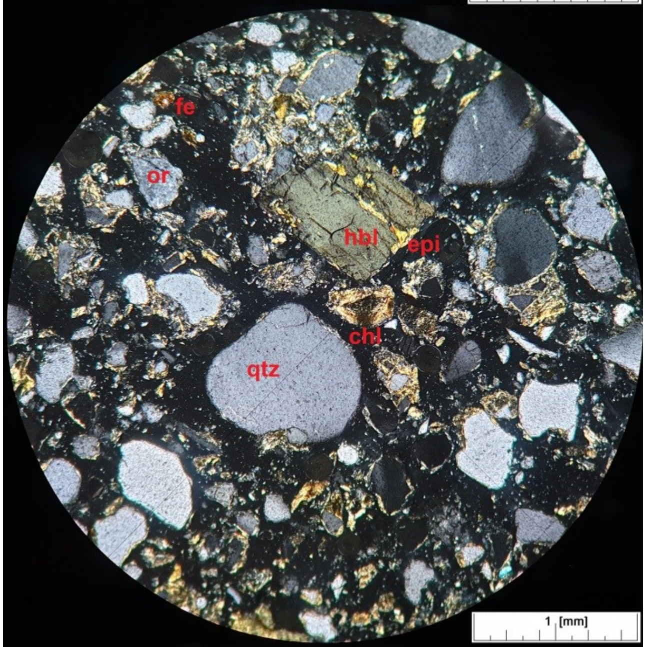

Quartz (qtz) orthoclase (or) with hornblende (hbl), epidote (epi), chlorite (chl) and iron compounds (fe).

j) LV499E (52˚22´N, 21˚48´E), d.: 25-45 cm

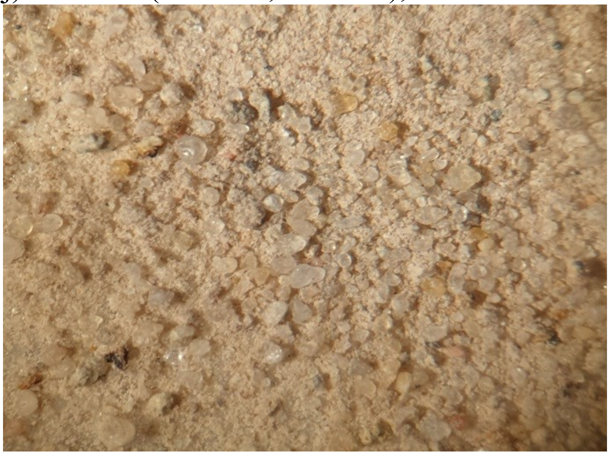

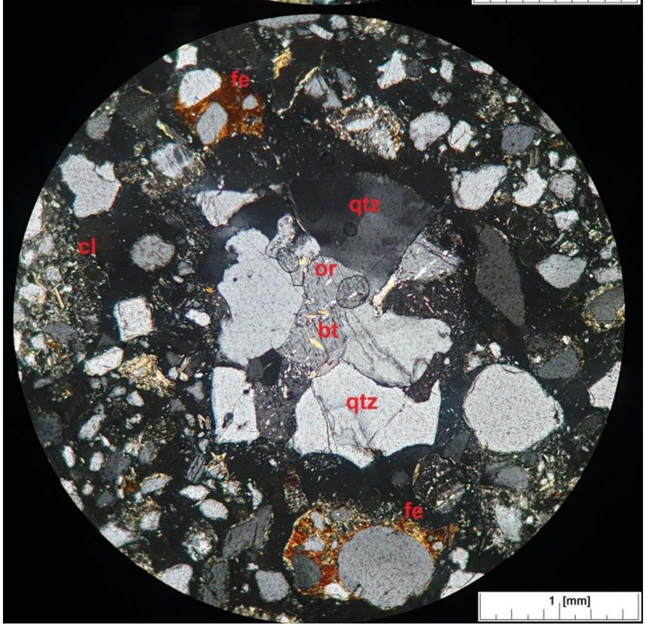

Quartz (qtz) with granite grains with orthoclase (or), clay minerals (cl), quartz with biotite (bt) and iron compounds (fe).

k) LV913Bt (50˚15´N, 21˚52´E), d.: 40-50 cm

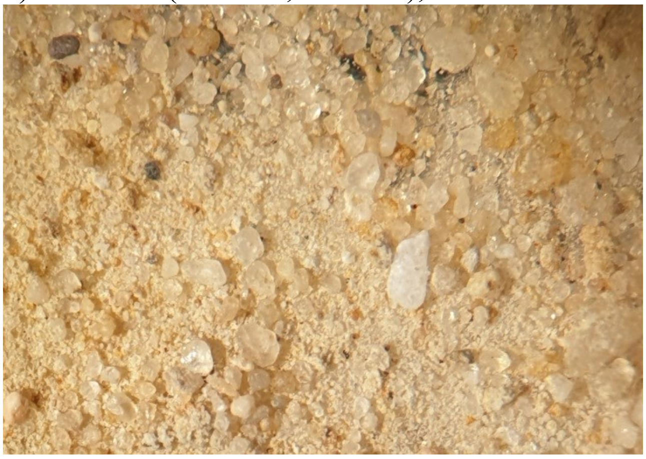

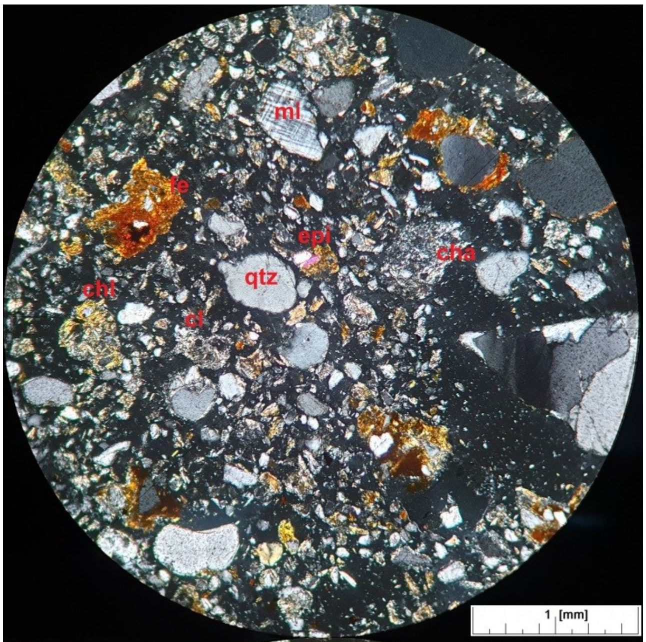

Quartz (qtz), microcline (mi), epidote (epi), chalcedony (cha), iron compounds (fe), chlorite (chl) and clay minerals (cl)

l) AR733C (50˚37´N, 19˚12´E), d.: 60-150 cm

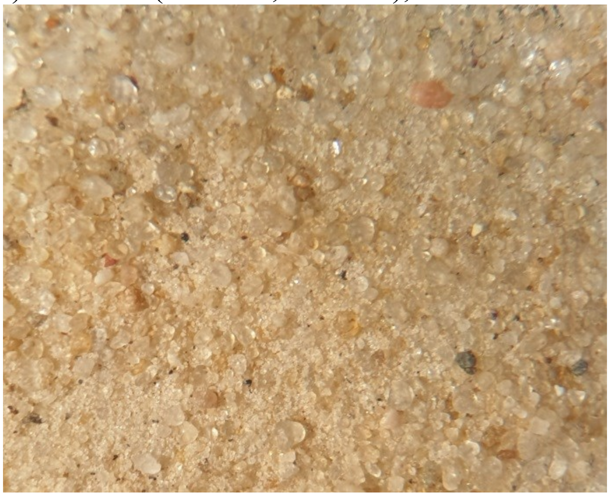

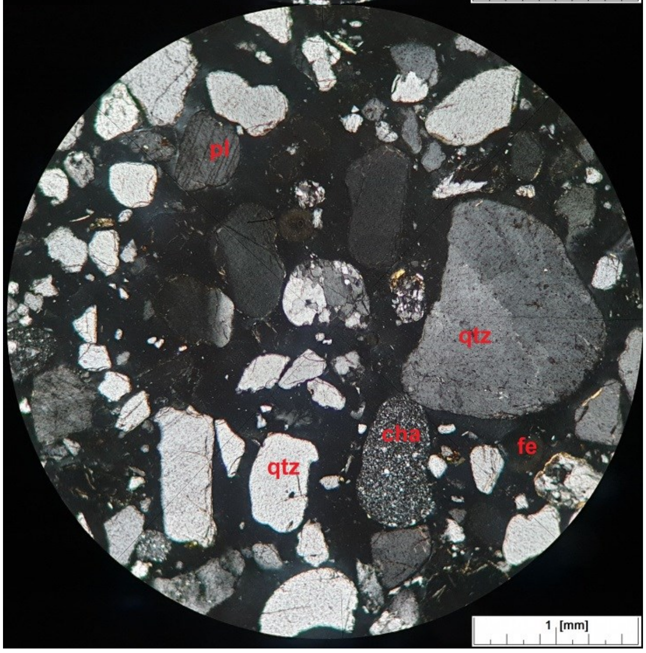

Quartz (qtz), chalcedonite (cha), plagioclase (pl) and limonite (goethite-lepidocrocite) (fe) around quartz crystalls.

S2 Fig. Photographs from the binocular magnifier (left side) and the polarizing optical microscope (thin sections, crossed polars, right side) of 12 selected Arenosols and Luvisols.

References

1. Bieganowski A, Witkowska-Walczak B, Gliński J, Sokołowska Z, Sławiński C, Brzezińska M, et al. Database of Polish arable mineral soils: a review. Int Agrophys. 2013;27:335-350. doi: 10.2478/intag-2013-0003.
2. ISO 11277. Soil quality - Determination of particle size distribution in mineral soil material - Method by sieving and sedimentation. 2009.

3. Paszko T. Adsorption, degradation and mobility of carbendazim in profiles of Polish mineral soils. Geoderma. 2014;226:160-169. doi: 10.1016/j.geoderma.2014.02.007.
4. Bertsch PM, Bloom PR. Aluminum. In: Sparks DL, editor. Methods of soil analysis Part 3 Chemical methods: Soil Science Society of America. Book Series no. 5. Agronomy; 1996. p. 517-543.
5. Mehra OP, Jackson ML. Iron oxide removal from soils and clays by a dithionite-citrate system buffered with sodium bicarbonate. Clay Clay Miner. 1960;7:317-327. doi: 10.1016/B978-0-08-009235-5.50026-7.
6. Pansu M, Gautheyrou J. Handbook of soil analysis. Berlin, Heidelberg, New York: Springer; 2006. 993 p.
