# Supplementary material for: Adsorption of bentazone in the profiles of mineral soils with low organic matter content
Source: PLoS One. 2020 Dec 2;15(12):e0242980. doi: 10.1371/journal.pone.0242980 (PMC7710104; doi:10.1371/journal.pone.0242980)
Supplement: S2 Appendix — S2 Table. Volumes of HCl, KCl, KOH and H2O used for the preparation of solutions used to stabilize pH and ionic strength during measurements the bentazone absorbance. S3 Table. Absorbance and pH of solutions measured to determine the pKa of bentazone. S4 Table. Results of fitting Eq (S1) to data presented in S2 Table. (PDF) [file pone.0242980.s002.pdf]

B Appendix. Determination the *pKa* of bentazone.

The value of pK_a has been determined spectrophotometrically using the modified method described by Albert and Serjeant [1]. A volume of 4.05 mL of 0.125 mM bentazone solution was mixed with 0.45 mL of the respective solution of HCl, KOH and KCl (S1 Table) and was stored in an incubator at 20°C for 60 min. The final concentration of bentazone in each of the duplicate samples was 0.1125 mM and ionic strength I = 0.01. The solutions from S1 Table were recommended by Albert and Serjeant [1] for organic acids with pK_a close to 2. At this pH, H_2O exhibits certain buffering capacity.

S2 Table. Volumes of HCl, KCl, KOH and H_2O used for the preparation of solutions used to stabilize pH and ionic strength during measurements the bentazone absorbance.

Solution No	1	2	3	4	5	6	7	8	9	10	11
V (mL) 0.1 M HCl	15.00	9.46	5.97	3.77	2.38	1.50	0.95	0.60	0.38	0.24	0
V (mL) 0.2 M KCl	0	2.77	4.51	5.62	6.31	6.75	7.03	7.20	7.31	7.38	7.31
V (mL) 0.1 M KOH	0	0	0	0	0	0	0	0	0	0	0.37
V (mL) H_2O	0	2.77	4.51	5.62	6.31	6.75	7.03	7.20	7.31	7.38	7.31

Then, the solution was transferred to a cuvette that was placed in the Varian Cary 50 Bio UV/VIS Spectrophotometer in which the cuvette temperature was maintained at 20°C by means of the Varian PCB 150 Water Peltier System. After 5 min, the absorbance was measured in triplicate for each sample at 333, 333.5, 334, 334.5, and 335 nm. Then, the pH of the solution was measured directly in the cuvette using a glass electrode. The obtained results are presented in S2 Table. Before pH measurements, the used pH-meter was calibrated with buffer standards with pH 1, 4, 7, and 9 ±0.05.

S3 Table. Absorbance and pH of solutions measured to determine the pK_a of bentazone.

Solution	Absorbance (A)[a]

No	pH[a]	333.0 nm	333.5 nm	334.0 nm	334.5 nm	335.0 nm
1a	2.144	0.0906	0.0883	0.0867	0.0845	0.0823
1b	2.134	0.0900	0.0876	0.0861	0.0839	0.0818
2a	2.345	0.1200	0.1178	0.1164	0.1143	0.1122
2b	2.337	0.1169	0.1150	0.1134	0.1114	0.1093
3a	2.530	0.1508	0.1489	0.1476	0.1458	0.1440
3b	2.520	0.1507	0.1490	0.1475	0.1458	0.1439
4a	2.721	0.1900	0.1884	0.1873	0.1856	0.1840
4b	2.723	0.1896	0.1881	0.1869	0.1853	0.1835
5a	2.967	0.2328	0.2316	0.2307	0.2293	0.2278
5b	2.975	0.2356	0.2344	0.2335	0.2320	0.2306
6a	3.180	0.2734	0.2724	0.2717	0.2705	0.2691
6b	3.155	0.2721	0.2712	0.2704	0.2692	0.2678
7a	3.366	0.3048	0.3041	0.3036	0.3025	0.3012
7b	3.353	0.3043	0.3036	0.3031	0.3020	0.3007
8a	3.576	0.3325	0.3320	0.3315	0.3307	0.3295
8b	3.566	0.3324	0.3319	0.3313	0.3304	0.3293
9a	3.795	0.3541	0.3537	0.3534	0.3526	0.3516
9b	3.769	0.3521	0.3519	0.3514	0.3508	0.3496
10a	4.013	0.3675	0.3674	0.3671	0.3663	0.3653
10b	3.972	0.3658	0.3656	0.3653	0.3646	0.3635
11a	7.593	0.3960	0.3961	0.3959	0.3954	0.3944
11b	7.547	0.3911	0.3911	0.3909	0.3905	0.3894

[a] mean of three measurements.

The pK_a value for bentazone was determined by fitting the equation describing the changes of absorbance connected with pH changes (Eq. (S1)) to the experimental data presented in S2 Table. Changes in absorbance of a weak monoprotic acid resulting from pH changes in a solution can be expressed as [1]:

$$\mathrm{A} = A_{an}\Phi_{an} + A_n\Phi_n \tag{S1}$$

where A_{an} is the absorbance maximum of an anionic form of the acid, and A_n is the absorbance maximum of a neutral form of the acid. The fractions of the anionic (Φ_{an}) and neutral forms of the weak monoprotic acid can be described using the commonly known expressions:

$$\Phi_{an} = {}^{1}/_{(1 + 10^{(pK_a - pH)})} \tag{S2}$$

$$\Phi_n = 1 - \Phi_{an} \tag{S3}$$

where $pK_a = - \log K_a$ and K_a is the dissociation constant of the acid. The dissociation constant values were determined separately for the absorbance measured at each of the five selected wavelengths (at which the changes of absorbance were the largest) by means of the nonlinear least squares method using the DataFit 9.1 (Oakdale Engineering) software. The results of fitting are presented in S3 Table.

S4 Table. Results of fitting Eq. (S1) to data presented in Table S2.

Absorbance	A_{an}	A_n	pK_a	SE[a]	R^2
333.0 nm	0.3914	0.0363	2.8612	0.0104	0.99951
333.5 nm	0.3914	0.0360	2.8614	0.0105	0.99951
334.0 nm	0.3912	0.0317	2.8613	0.0103	0.99952
334.5 nm	0.3907	0.0293	2.8617	0.0104	0.99951
335.0 nm	0.3897	0.0268	2.8608	0.0104	0.99951

[a] standard error for pK_a.

The average value of the *pKa* from S3 Table is 2.861 and mean standard error (SE) of the five estimates is 0.010. According to Albert and Serjeant [1], for experiments carried out at 20°C and at I = 0.01, the obtained mixed type of K_a for the monoprotic acid can be converted to the thermodynamic dissociation constant K_a^T using the following equation:

$$pK_a^T = pK_a + \frac{0.507\sqrt{I}}{1+1.5\sqrt{I}} \tag{S4}$$

The obtained pK_a^T value was 2.905, and the recalculated standard error was again 0.010. It is worth mentioning that the above pK_a^T value is very similar to the value of $pK_a^T = 2.92 \pm 0.06$ obtained at 20°C by Comer et al. [2] by means of the pH-metric technique based on potentiometric titration. To the best of our knowledge, other experimentally determined pK_a values for bentazone are not available in the literature.

References

1. Albert A, Serjeant EP. The determination of ionization constants. A laboratory manual. Third edition. London, New York: Chapman and Hall; 1984. 218 p.
2. Comer J, Chamberlain K, Evans A. Validation of pH-metric technique for measurement of pKa and log Pow of ionizable herbicides. SAR and QSAR in Environmental Research. 1995;3(4):307-313. doi: 10.1080/10629369508050157.
