# Supplementary material for: Adsorption of bentazone in the profiles of mineral soils with low organic matter content
Source: PLoS One. 2020 Dec 2;15(12):e0242980. doi: 10.1371/journal.pone.0242980 (PMC7710104; doi:10.1371/journal.pone.0242980)
Supplement: S3 Appendix — S5 Table. Mineralogical composition (%) of the 12 soils selected from S1 Table with pH in 0.01 M CaCl2 < 5.0 and Coc < 0.35%. S3 Fig. Sample diffractograms of soil samples from 76 (NW Poland), 611 (CE Poland) and 872 (SE Poland) profiles. Acronyms denote: bt–biotite, cl–clinochlore, epi–epidote, gl–glauconite, il–illite, ka–kaolinite, mu–muscovite, or–orthoclase, qzt–quartz, ref–CaF2, and ru–rutile. (PDF) [file pone.0242980.s003.pdf]

C Appendix. Sample preparation and X-ray powder diffraction.

Before measurement soil samples were ground in a Retsch MM200 Oscillating Mill Grinder for 30 min with a frequency of 28 Hz in 10 mL grinding jars filled with 3 stainless steel balls (4 mm diameter). The 30 min milling time was a compromise between the finer powder necessary for precise quantitative analysis and the crystal grain quality. 0.8 g of each sample was then weighted and 0.2 g of CaF_2 (extra pure 99.9%; Laboratory BDH reagents, England) was added as an internal standard according to Klug and Alexander [1]. Such prepared samples were analyzed using a powder X-ray diffraction technique with an Empyrean, Malvern Panalytical diffractometer with a Cu anode as a source of CuKα X-ray radiation (λ =1.5406 Å), soller slits, antiscatter detector slit, and an X-ray mirror. All samples were measured over a 2θ range of 4 to 90º with a step size of 0.006º with an exposition time per step of 1.135 s. During measurement samples were spun at 8 rpm. The temperature during measurement was 20ºC. X-ray diffraction data were fitted using ReX v.0.91 Rietveld analysis software [2]. All necessary crystal structure files (cif) were downloaded from the American Mineralogist Crystal Structure Data Base [3] based on results of microscopic and elemental analysis of soil grains.

During fitting all diffraction specific parameters like wave length, goniometer radius and sample detector distance were fixed. The reflections FWHM (described by domain size) of quartz and CaF_2 were tested independently and fixed (Quarts – $1.277x10^6$, CaF_2 – 320). Isotropic parameters in most cases were taken from original cif files if were measured at 20-25º C or set to 0.05 $Å^2$. The following parameters were adjusted for each sample: scale factor, 2-theta offset, all polynomial coefficients for background, volume fraction and domain size. In the fitting process orthoclase was represented by 3 models with slightly change unit constants to obtain best fit the observed reflections. The pseudo-Voight profile function described best the reflections. The fitting procedure was repeated until R_1, which indicates final agreement between the observed and calculated patterns, was smaller than 0.12. The detection limit was 0.01 g and the relative standard deviation of the repeatability of the results ~4% (< 4% for minerals which content in a soil sample was > 5%, and > 4% for minerals which content was < 1%).

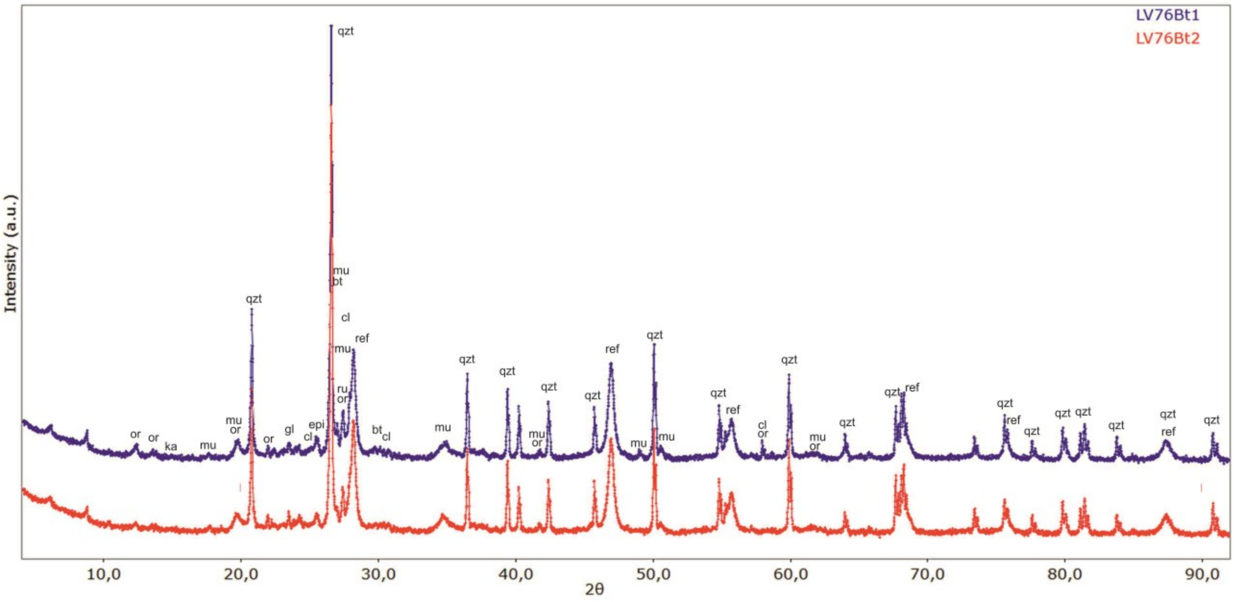
LV76Bt1
LV76Bt2
Intensity (a.u.)
2θ

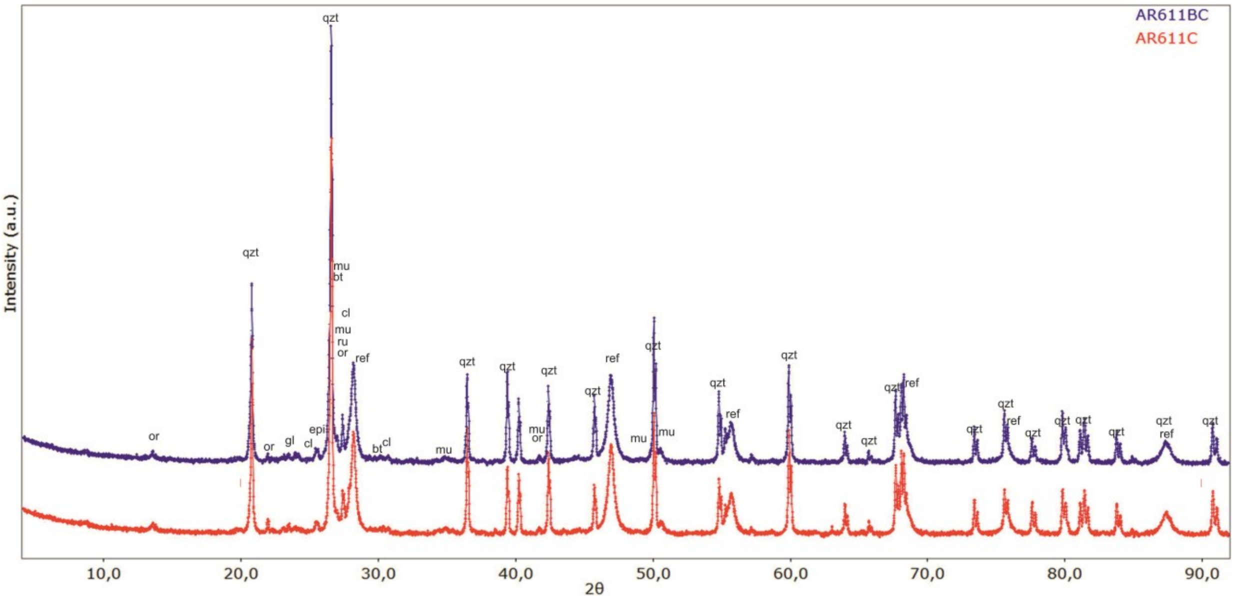
AR611BC
AR611C
Intensity (a.u.)
2θ

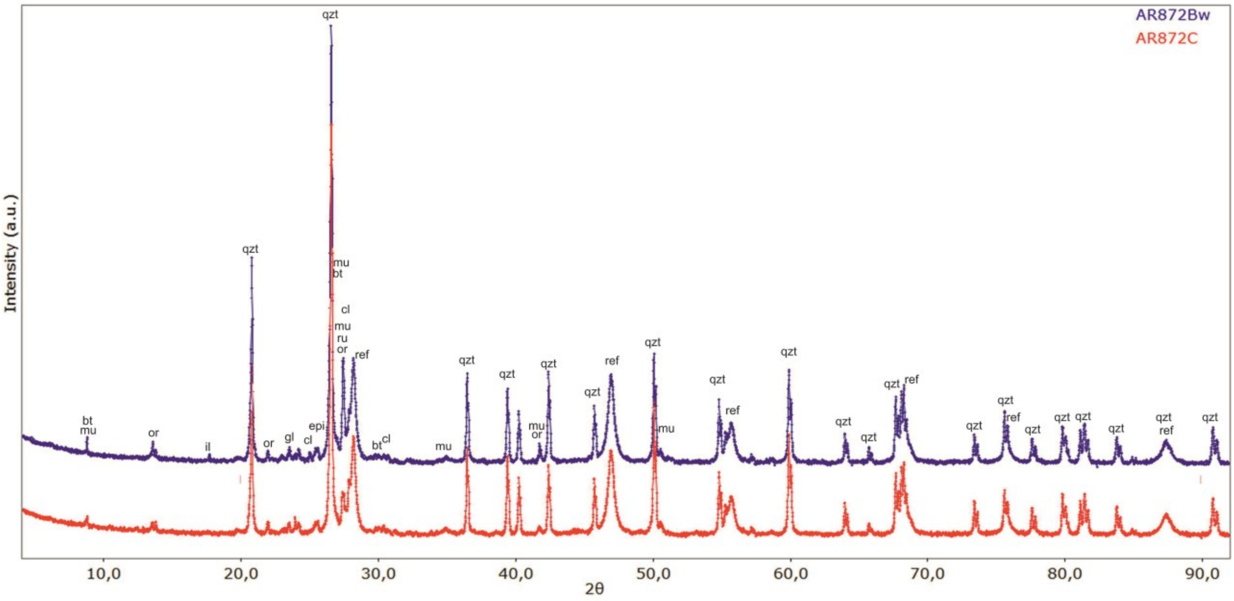
AR872Bw
AR872C
Intensity (a.u.)
2θ

S3 Fig. Sample diffractograms of soil samples from 76 (NW Poland), 611 (CE Poland) and 872 (SE Poland) profiles. Acronyms denote: bt – biotite, cl – clinochlore, epi – epidote, gl – glauconite, il – illite, ka – kaolinite, mu – muscovite, or – orthoclase, qzt – quartz, ref – CaF_2, and ru – rutile.

S5 Table. Mineralogical composition (%) of the 12 soils selected from S1 Table with pH in 0.01 M $CaCl_2$ < 5.0 and C_{oc} < 0.35%.

Soil	Quartz	Orthoclase	Rutile	Muscovite	Illite	Kaolinite	Epidote	Titanite	Albite	Biotite	Glauconite	Clinochlore	Vermiculite	Lepidocrocite	Diopside	Amorphous[a]
AR528BC	77.85	8.28	9.55	1.36	0.90	Nd[b]	0.62	nd	0.35	nd	nd	0.27	nd	nd	0.07	0.76
AR281Bw	76.39	9.16	3.64	0.63	0.14	nd	0.43	nd	0.29	nd	nd	0.20	nd	0.11	nd	9.00
AR872Bw	76.47	8.57	12.67	nd	nd	nd	nd	0.48	0.41	nd	nd	nd	nd	nd	nd	0.85
AR611BC	82.43	5.06	3.23	0.69	nd	nd	0.19	nd	0.17	nd	nd	nd	nd	nd	nd	8.22
LV50E	71.24	10.52	2.67	3.22	0.55	0.65	0.59	nd	0.23	0.36	0.47	0.25	nd	0.11	nd	8.83
LV76Bt1	60.90	14.39	2.83	5.08	1.78	1.24	0.64	1.02	0.24	0.58	0.23	0.19	0.63	0.13	nd	9.89
LV499E	69.13	13.26	4.47	3.46	0.72	0.61	0.66	nd	0.29	0.58	0.37	0.30	nd	0.12	nd	5.69
LV913Bt	55.60	8.11	2.62	5.24	2.37	1.30	0.10	nd	0.11	0.55	0.20	0.14	0.34	0.16	nd	23.17
AR733C	80.17	6.21	4.56	0.72	nd	0.04	0.15	nd	0.12	nd	nd	0.17	nd	0.06	nd	7.80
AR872C	79.69	10.10	5.50	1.12	nd	0.01	0.30	0.75	0.51	nd	nd	0.21	nd	nd	nd	1.36
AR611C	86.49	9.10	1.68	0.29	nd	0.03	0.37	1.04	0.34	nd	nd	0.16	nd	0.08	0.01	0.37
LV76Bt2	59.02	12.00	2.20	4.54	2.38	1.04	0.25	0.76	0.14	0.90	0.80	0.17	0.85	0.07	nd	14.69

[a] amorphous phase and minerals below the detection limit; [b] amount below the detection limit.

References

1. X-ray diffraction procedures for polycrystalline and amorphous materials [Internet]. John Wiley & Sons. 1974.
2. Bortolotti M, Lutterotti L, Lonardelli I. ReX: a computer program for structural analysis using powder diffraction data. J Appl Cryst. 2009;42:538-539. doi: 10.1107/S0021889809008309.
3. Downs RT, Hall-Wallace M. The American Mineralogist Crystal Structure Database. Amer Miner. 2003;88:247-250.
