# Supplementary material for: Adsorption of bentazone in the profiles of mineral soils with low organic matter content
Source: PLoS One. 2020 Dec 2;15(12):e0242980. doi: 10.1371/journal.pone.0242980 (PMC7710104; doi:10.1371/journal.pone.0242980)
Supplement: S4 Appendix — S4 Fig. The results of the batch kinetic experiments. S6 Table. The results of fitting Eq (S5) to the batch experiment data. S7 Table. Kd values obtained from batch experiments for soils from S1 Table. (PDF) [file pone.0242980.s004.pdf]

D Appendix. Adsorption kinetics and K_d values from batch experiments.

The kinetics of bentazone adsorption on the soil solid phase has been described using the model assuming initial instantaneous adsorption, expressed by the S_{Inst} (µg/g), and next the time-dependent adsorption step described by the first-order coefficient k_a, which finished at adsorption equilibrium expressed by the S_{Eq} (µg/g) coefficient [1] :

$$S_t = S_{Inst} + (S_{Eq} - S_{Inst})\,(1-exp(-k_a t)) \qquad \text{(S5)}$$

The results of fitting are presented in S6 Table and in S4 Fig.

S6 Table. The results of fitting Eq. (S5) to the batch experiment data.

Soil	S_{Inst} (µg/g)	S_{Eq} (µg/g)	k_a (h^{-1})	RSS
611 Ap	0.137	0.160	0.330	0.0005
611 BC	0.016	0.038	0.774	0.0004
611 C	0.004	0.052	1.851	0.0002
590 Ap	0.140	0.203	0.281	0.0007
590 E	0.014	0.026	0.566	0.0013
590 Bt	0.002	0.015	0.544	0.0005
564 Ap	0.097	0.153	0.446	0.0010
564 Bw	0.058	0.091	0.177	0.0007
564 BC	0.023	0.085	0.472	0.0009

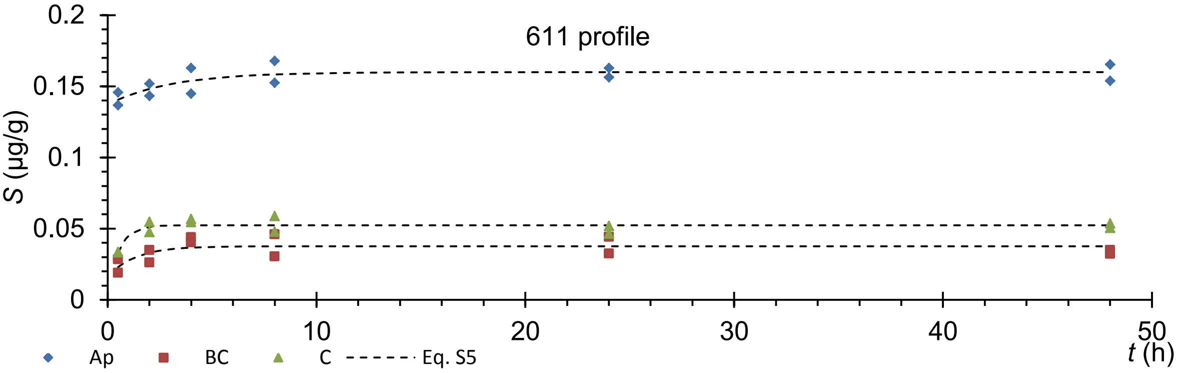

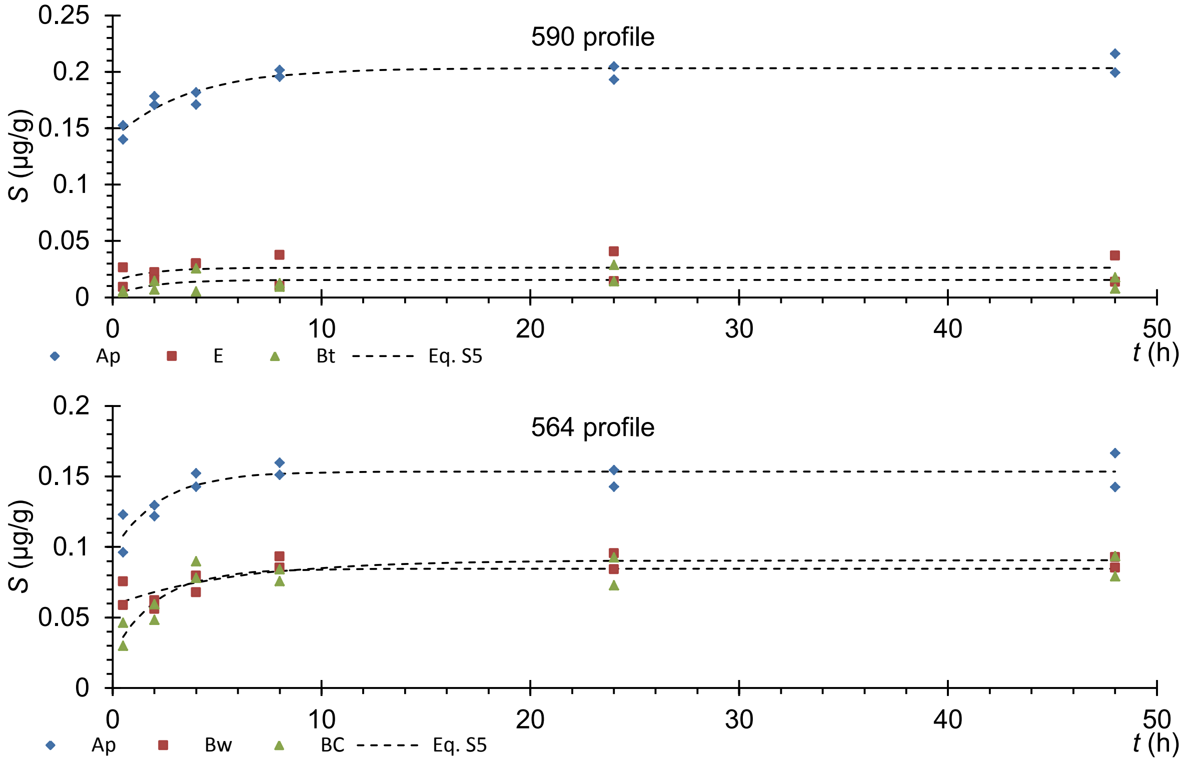

S4 Fig. The results of the batch kinetic experiments.

S7 Table. K_d values obtained from batch experiments for soils from S1 Table.

Profile	Ap (topsoil)			B (upper subsoil)			C (lower subsoil)		
	K_d (mL/g)	SD	pH	K_d (mL/g)	SD	pH	K_d (mL/g)	SD	pH
Arenosols (AR)									
45	0.108	0.002	4.75	0.011	0.004	5.89	0.006	0.001	5.49
119	0.165	0.004	4.4	0.038	0.005	5.65	0.013	0.001	5.37
232	0.210	0.007	3.99	0.037	0.005	5.19	0.007	0.002	5.28
281	0.305	0.005	3.69	0.037	0.011	4.96	0.021	0.004	4.97
297	0.119	0.015	4.42	0.013	0.005	5.60	0.007	0.001	5.62
360	0.087	0.016	4.87	0.010	0.003	6.16	0.003	0.002	6.66
528	0.107	0.003	5.14	0.027	0.002	4.89	0.016	0.002	5.12
611	0.145	0.016	4.16	0.031	0.001	4.66	0.040	0.002	4.52
733	0.114	0.004	4.88	0.062	0.001	4.62	0.050	0.002	4.40
774	0.145	0.002	4.27	0.033	0.003	5.23	0.001	0	6.14
872	0.172	0.016	4.00	0.035	0.004	4.80	0.062	0.001	4.30
Luvisols (LV)									
50	0.088	0.011	5.13	0.042	0.003	4.71	0.009	0.002	5.35
76	0.096	0.002	5.33	0.024	0.003	4.81	0.025	0.003	4.58
204	0.127	0.002	6.15	0.024	0.003	6.44	0.007	0.004	6.74

341	0.094	0.004	6.77	0.017	0.002	6.86	0.013	0.001	6.23
348	0.066	0.022	5.53	0.010	0.002	6.65	0.010	0.006	6.43
499	0.236	0.04	4.21	0.028	0.003	4.86	0.021	0.001	5.17
590	0.093	0.009	4.86	0.010	0.002	5.34	0.006	0.001	5.23
625	0.068	0.016	5.41	0.011	0.001	6.58	0.015	0	5.29
913	0.107	0.005	4.50	0.025	0.006	4.69	0.011	0.002	5.37
Luvisols or Cambisols (LV&CM)									
564	0.052	0.014	6.77	0.028	0.002	6.90	0.033	0.01	6.63
662	0.097	0.024	7.03	0.071	0.004	6.03	0.020	0.003	5.59
713	0.095	0.001	6.85	0.017	0.004	6.61	0.011	0.004	5.18
788	0.082	0.014	7.11	0.037	0.005	7.43	0.039	0.004	7.73
795	0.072	0.003	5.42	0.019	0.005	6.92	0.031	0.003	7.79
886	0.087	0.008	5.48	0.016	0.006	6.85	0.012	0.002	6.82
898	0.100	0.003	5.37	0.018	0.004	5.53	0.014	0.003	5.05

References

1. Paszko T, Jankowska M. Modeling the effect of adsorption on the degradation rate of propiconazole in profiles of Polish Luvisols. Ecotoxicology and environmental safety. 2018;161:584-593. doi: 10.1016/j.ecoenv.2018.05.093.
