# Supplementary material for: Adsorption of bentazone in the profiles of mineral soils with low organic matter content
Source: PLoS One. 2020 Dec 2;15(12):e0242980. doi: 10.1371/journal.pone.0242980 (PMC7710104; doi:10.1371/journal.pone.0242980)
Supplement: S5 Appendix — S8 Table. Kendall rank correlation matrix for soil properties and Kd (n = 81 soils). (PDF) [file pone.0242980.s005.pdf]

E Appendix. Correlations between soil properties and K_d.

S8 Table. Kendall rank correlation matrix for soil properties and K_d (n = 81 soils).

	K_d	C_{oc}	pH	C_{sand} (2000-50)	$C_{vcos+cos}$ (2000-500)	C_{ms} (500-250)	C_{fs+vfs} (250-50)	C_{silt} (50-2)	C_{cosi} (50-20)	C_{fsi} (20-2)	C_{clay} (<2)	C_{Al}	C_{Fe}
C_{oc}	0.644												
	<0.001												
pH	-0.306	-0.011											
	<0.001	*0.890*											
C_{sand} (2000-50)	-0.014	-0.256	-0.318										
	0.858	*0.001*	*<0.001*										
$C_{vcos+cos}$ (2000-500)	0.112	-0.042	-0.343	0.437									
	0.151	*0.596*	*<0.001*	*<0.001*									
C_{ms} (500-250)	0.121	-0.113	-0.425	0.696	0.695								
	0.119	*0.143*	*<0.001*	*<0.001*	*<0.001*								
C_{fs+vfs} (250-50)	0.035	-0.132	-0.311	0.680	0.149	0.377							
	0.642	*0.082*	*<0.001*	*<0.001*	*0.056*	*<0.001*							
C_{silt} (50-2)	0.053	0.300	0.332	-0.925	-0.412	-0.658	-0.649						
	0.483	*<0.001*	*<0.001*	*<0.001*	*<0.001*	*<0.001*	*<0.001*						
C_{cosi} (50-20)	-0.001	0.236	0.356	-0.863	-0.415	-0.667	-0.611	0.870					
	0.987	*0.002*	*<0.001*	*<0.001*	*<0.001*	*<0.001*	*<0.001*	*<0.001*					
C_{fsi} (20-2)	0.072	0.329	0.322	-0.886	-0.398	-0.630	-0.624	0.925	0.795				
	0.344	*<0.001*	*<0.001*	*<0.001*	*<0.001*	*<0.001*	*<0.001*	*<0.001*	*<0.001*				
C_{Clay} (<2)	-0.178	0.003	0.199	-0.609	-0.242	-0.469	-0.385	0.533	0.543	0.520			
	0.019	*0.971*	*0.009*	*<0.001*	*0.002*	*<0.001*	*<0.001*	*<0.001*	*<0.001*	*<0.001*			
C_{Al}	0.293	0.054	-0.751	0.198	0.280	0.334	0.212	-0.210	-0.227	-0.213	-0.155		
	<0.001	*0.481*	*<0.001*	*0.009*	*<0.001*	*<0.001*	*0.005*	*0.006*	*0.003*	*0.005*	*0.041*		
C_{Fe}	0.307	0.198	-0.333	0.201	0.261	0.274	0.262	-0.186	-0.218	-0.172	-0.152	0.271	
	<0.001	*0.009*	*<0.001*	*0.008*	*0.001*	*<0.001*	*0.001*	*0.014*	*0.004*	*0.023*	*0.045*	*<0.001*	
C_{Mn}	0.332	0.380	-0.202	-0.117	0.022	-0.034	-0.002	0.135	0.088	0.168	0.067	0.190	0.127
	<0.001	*<0.001*	*0.008*	*0.121*	*0.778*	*0.663*	*0.981*	*0.075*	*0.247*	*0.027*	*0.374*	*0.012*	*0.093*
