# Supplementary material for: Adsorption of bentazone in the profiles of mineral soils with low organic matter content
Source: PLoS One. 2020 Dec 2;15(12):e0242980. doi: 10.1371/journal.pone.0242980 (PMC7710104; doi:10.1371/journal.pone.0242980)
Supplement: S6 Appendix — S9 Table. Kd values for bentazone available in literature. (PDF) [file pone.0242980.s006.pdf]

F Appendix. Comparison of K_d values available in literature.

S9 Table. K_d values for bentazone available in literature.

Author	Solution during pH determination	Solution during adsorption	Soil/solution ratio	Measurement method	Remarks	Soil name	C_{oc} (%)	C_{sand} (%)	pH value	K_d (mL/g)
Bonfleur et al. [1]	$CaCl_2$ solution	0.01 M $CaCl_2$	5 g/5 mL	^{14}C	As $K_{d\ obs.}$	LV	4.8	5.6	5	0.5
					$K_{d\text{-}exp}$ from Table 4	LB	4	8	5.5	0.16
					have	LB1	4.7	4.6	5.2	0.3
					been	LV1	3.7	4.3	4.9	0.27
					used	LVA	3.2	37.9	4.6	0.25
						LVA1	2	67.79	4.6	0.14
						LV7	2.4	30.7	4.5	0.27
						LV8	3	19.5	5.4	0.12
						LV9	3.7	11	5.8	0.14
						LV11	2.1	51.12	5.5	0.13
						LV12	2.3	40.39	5.6	0.14
Gaston et al. [2]	0.01 M $CaCl_2$ (1:1)	0.01 M $CaCl_2$	5 g/15 mL	^{14}C	Lack data for	NT 0-10	1.02	n/a	5.6	0.039
					C_{sand} but soil	NT 10-20	0.56		5.26	0.039
					pH > 5	NT 20-30	0.44		5.71	0.039
						CT 0-10	0.87		5.79	0.039
						CT 10-30	0.64		5.77	0.039
						CT 20-30	0.49		5.8	0.039
Gaston et al. [3]	n/a	0.01 M $CaCl_2$	2.5 g/7.5 mL	^{14}C		Dundee	0.87	n/a	5.79	0.03
Li et al. [4]	H_2O (1:1)	0.01 M $CaCl_2$	10 g/15 mL	HPLC	C_{oc} calculated	1	2.32	6.1	7.63	0.206
					from C_{OM}	2	0.37	15.6	4.15	0.162
						3	2.49	34.4	7.28	0.140
						4	1.62	29.3	4.84	0.233
						5	2.32	11.3	4.86	0.321

Madsen et al. [5]	H_2O (1:1)	H_2O	5 g/5 mL	^{14}C	10°C	1	0.05	99	4.9	0.05
						2	0.03	99	5.2	0.02
						3	0.02	98	4.7	0.005
						4	0.02	100	6.6	0.005
						5	0.03	~100	7.5	0.02
						6	0.07	94	7.7	0.02
						7	0.06	96	7.8	0.02
						8	0.12	81	7.8	0.01
						9	0.08	90	7.8	0.020
						10	0.12	62	7.8	0.005
Rodríguez-Cruz et al. [6]	H_2O (1:2.5)	0.01 M $CaCl_2$	5 g/10 mL	HPLC	C_{oc} calculated from C_{OM}	Topsoil	1.67	68.7	6.69	0.06
						Subsoil	0.92	65.3	7.42	0.11
Rodríguez-Cruz et al. [7]	H_2O (1:2.5)	0.01 M $CaCl_2$	5 g/10 mL	HPLC	Lack data for C_{sand} but soil pH > 5	0-10	1.52	n/a	7.06	0.15
						20-30	1.35		7.13	0.11
						40-50	1.15		7.79	0.10
						60-70	0.96		7.98	0.09
						70-80	0.78		8.09	0.06

References

1. Bonfleur EJ, Kookana RS, Tornisielo VL, Regitano JB. Organomineral interactions and herbicide sorption in Brazilian tropical and subtropical Oxisols under no-tillage. J Agric Food Chem. 2016;64:3925-3934. doi: 10.1021/acs.jafc.5b04616.
2. Gaston LA, Locke MA, Zablotowicz RM. Sorption and degradation of bentazon in conventional- and no-till Dundee soil. J Environ Qual. 1996;25:120-126. doi: 10.2134/jeq1996.00472425002500010016x.
3. Gaston LA, Locke MA, Wagner SC, Zablotowicz RM, Reddy KN. Sorption of bentazon and degradation products in two Mississippi soils. Weed Sci. 1996;44:678-682. doi: 10.1017/S0043174500094522.
4. Li K, Liu W, Xu D, Lee S. Influence of organic matter and pH on bentazone sorption in soils. J Agric Food Chem. 2003;51:5362-5366. doi: 10.1021/jf0343332.
5. Madsen L, Lindhardt B, Rosenberg P, Clausen L, Fabricius I. Pesticide sorption by low organic carbon sediments: a screening for seven herbicides. J Environ Qual. 2000;29:1488-1500. doi: 10.2134/jeq2000.00472425002900050016x.
6. Rodríguez-Cruz MS, Jones JE, Bending GD. Field-scale study of the variability in pesticide biodegradation with soil depth and its relationship with soil characteristics. Soil Biol Biochem. 2006;38(9):2910-2918. doi: 10.1016/j.soilbio.2006.04.051.
7. Rodríguez-Cruz MS, Jones JE, Bending GD. Study of the spatial variation of the biodegradation rate of the herbicide bentazone with soil depth using contrasting incubation methods. Chemosphere. 2008;73(8):1211-1215. doi: 10.1016/j.chemosphere.2008.07.044.
