# Supplementary material for: Adsorption of bentazone in the profiles of mineral soils with low organic matter content
Source: PLoS One. 2020 Dec 2;15(12):e0242980. doi: 10.1371/journal.pone.0242980 (PMC7710104; doi:10.1371/journal.pone.0242980)
Supplement: S7 Appendix — S5 Fig. The point of zero net proton charge (PZNPC) of the < 500 μm AR774C fraction. (PDF) [file pone.0242980.s007.pdf]

G Appendix. Determination of the point of zero net proton charge.

The soil from the C horizon of 774 profile of AR was sieved through a 500 µm sieve. The 2 g samples of the sieved soil were weight to 30 mL polypropylene tubes and 20 mL of respective solution was dosed. The pH values of used solutions were adjusted using 0.01 M HCl or NaOH and their ionic strength by adding NaCl as indifferent electrolyte. The three series of samples with NaCl concentrations of 0.0025, 0.05 and 0.15 M were used. The screwed tubes with the soil and the blank samples (tubes with solution but with no soil) were agitated for 48 h. Next pH in each soil sample (pH_S) and the respective blank sample (pH_B) was measured. Based on the measured pH_S and pH_B the apparent proton surface charge density ($\sigma_{H,titr}$) for a given pH was calculated using the following equation:

$$\sigma_{H,titr} = M_{soln}\left\{([H^+]_B - [H^+]_S) - \left(\frac{K_W}{[H^+]_B} - \frac{K_W}{[H^+]_S}\right)\right\} \tag{S6}$$

where M_{soln} is the mass of the electrolyte solution equilibrated with the soil per unit adsorbent mass. $[H^+]$ is the solution proton concentration (mol/kg), $K_w = 10^{-14}$ (mol^2/kg^2) is the dissociation constant in water, and the subscripts S and B refer to sample and blank, respectively. The net proton surface-charge density (σ_H) was calculated from:

$$\sigma_H = \sigma_{H,titr} - \langle\sigma_{H,titr}(PZNPC)\rangle_{ave} \tag{S7}$$

where $\langle\sigma_{H,titr}(PZNPC)\rangle_{ave}$ is the average value (for the three measured ionic strengths) of the apparent proton surface charge density at the point of the zero net proton charge (PZNPC), a constant independent of pH, estimated according to Schroth and Sposito [1].

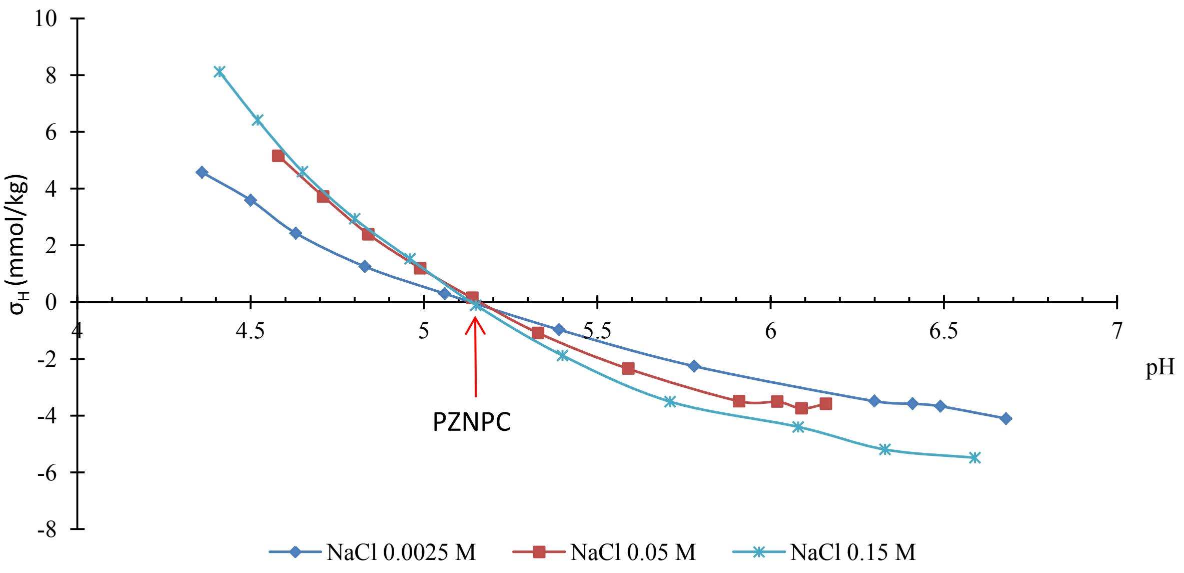

Fig. S5. The point of zero net proton charge (PZNPC) of the < 500 µm AR774C fraction.

References

1. Schroth BK, Sposito G. Surface charge properties of kaolinite. Clay Clay Miner. 1997;45(1):95-91. doi: 10.1557/PROC-432-87.
