# Supplementary material for: Adsorption of bentazone in the profiles of mineral soils with low organic matter content
Source: PLoS One. 2020 Dec 2;15(12):e0242980. doi: 10.1371/journal.pone.0242980 (PMC7710104; doi:10.1371/journal.pone.0242980)
Supplement: S8 Appendix — S6 Fig. Fractions of AR611C –photographs from the binocular magnifier showing quartz, orthoclase, chalcedonite, glauconite and accessory minerals. S7 Fig. Backscattered scanning electron (BSE) micrographs of the AR611C grains: fraction 150–63 μm (a), typical quartz grain surface with v-shaped holes and adsorbed clay minerals (b). S8 Fig. Diffractograms of fractions of AR611C. Acronyms denote: bt–biotite, cl–clinochlore, epi–epidote, gl–glauconite, mu–muscovite, or–orthoclase, qzt–quartz, ref–CaF2 and ru–rutile. S10 Table. Results of adsorption experiments using the fractions obtained by sieving the native AR611C soil. S11 Table. Properties of AR611C fractions used for the adsorption experiments. (PDF) [file pone.0242980.s008.pdf]

H Appendix. Adsorption in fractions of AR611C.

S10 Table. Results of adsorption experiments using the fractions obtained by sieving the native AR611C soil.

Fraction (µm)	K_d (mL/g)	SD	pH
2000-500	0.022	0.003	4.99
500-400	0.012	0.003	5.16
400-150	0.033	0.010	4.81
150-63	0.071	0.012	4.56
63-40	0.099	0.008	4.49
< 40	0.089	0.010	4.62

a) 2000-500 µm

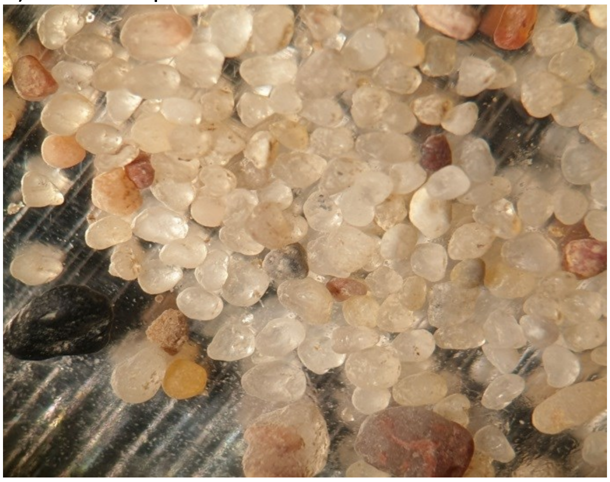

b) 500-400 µm

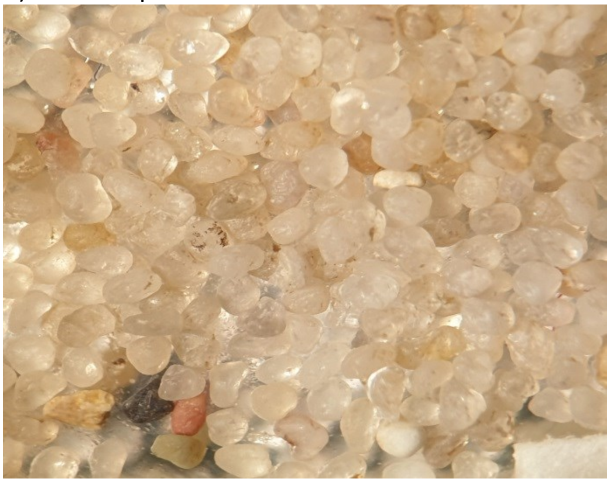

c) 400-150 µm

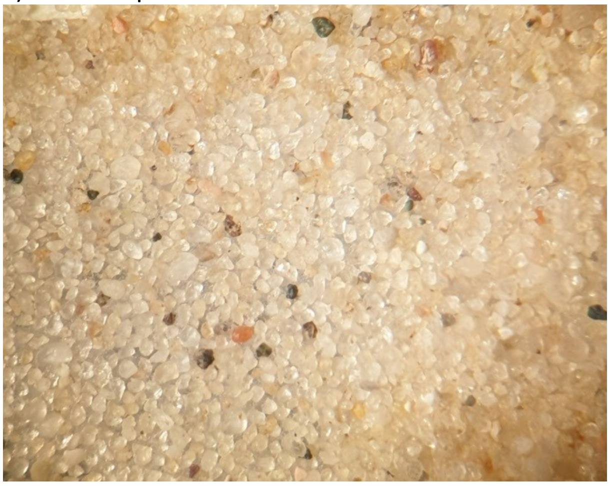

d) 150-63 µm

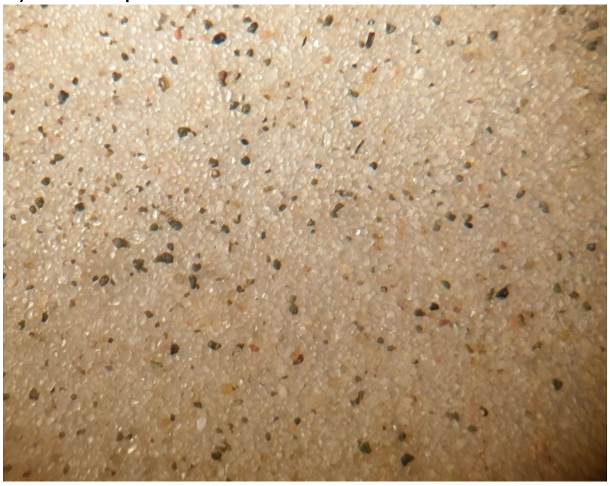

e) 63-40 µm

f) < 40 µm

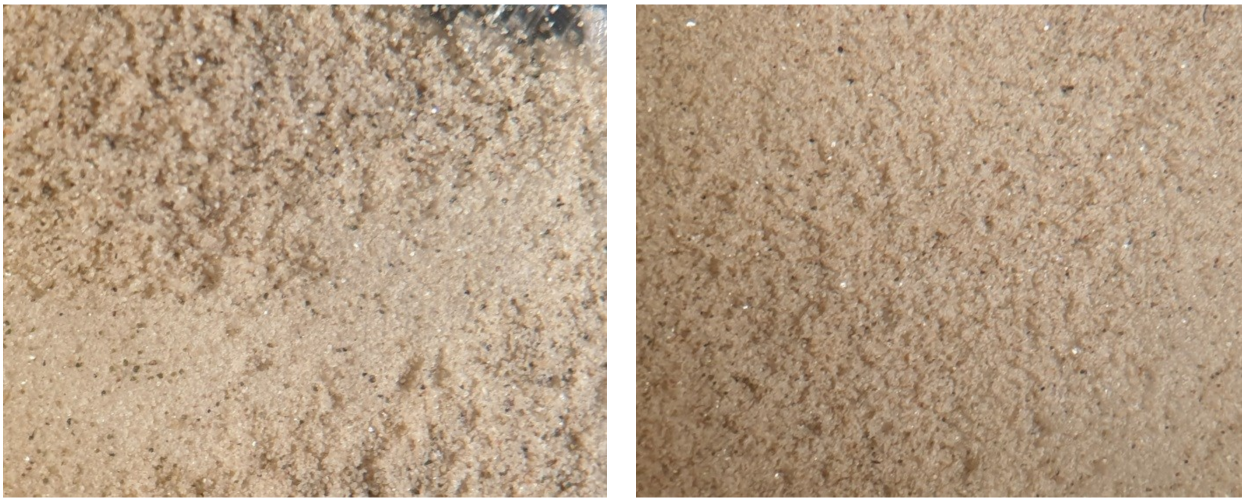

S6 Fig. Fractions of AR611C – photographs from the binocular magnifier showing quartz, orthoclase, chalcedonite, glauconite and accessory minerals.

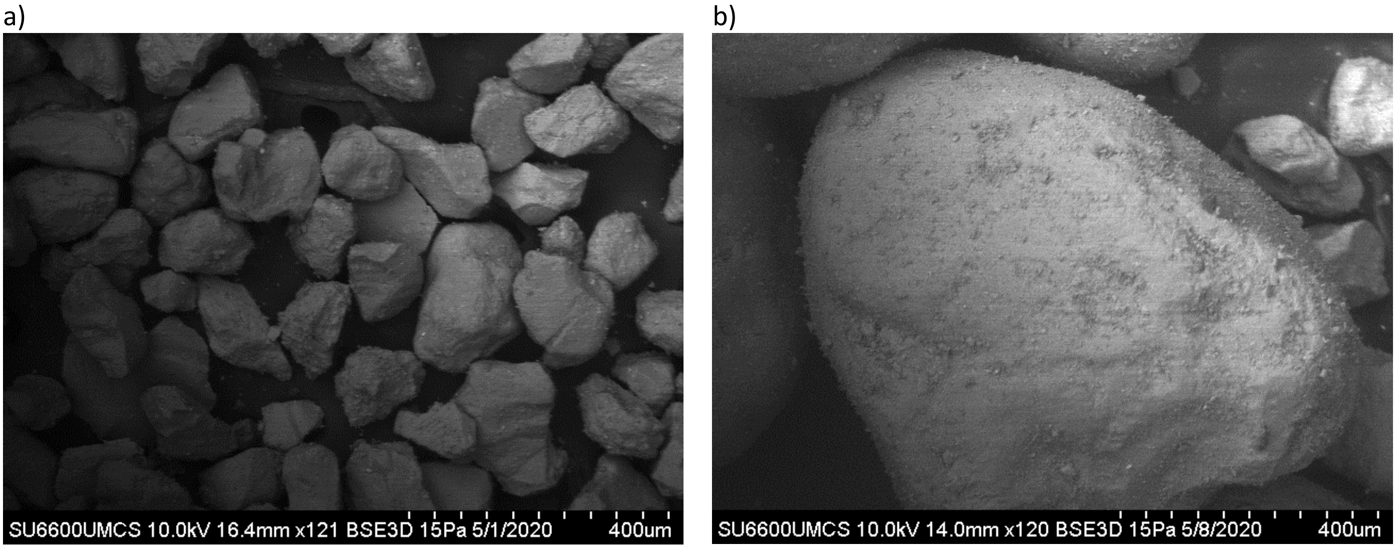

S7 Fig. Backscattered scanning electron (*BSE*) micrographs of the AR611C grains: fraction 150-63 μm (a), typical quartz grain surface with v-shaped holes and adsorbed clay minerals (b).

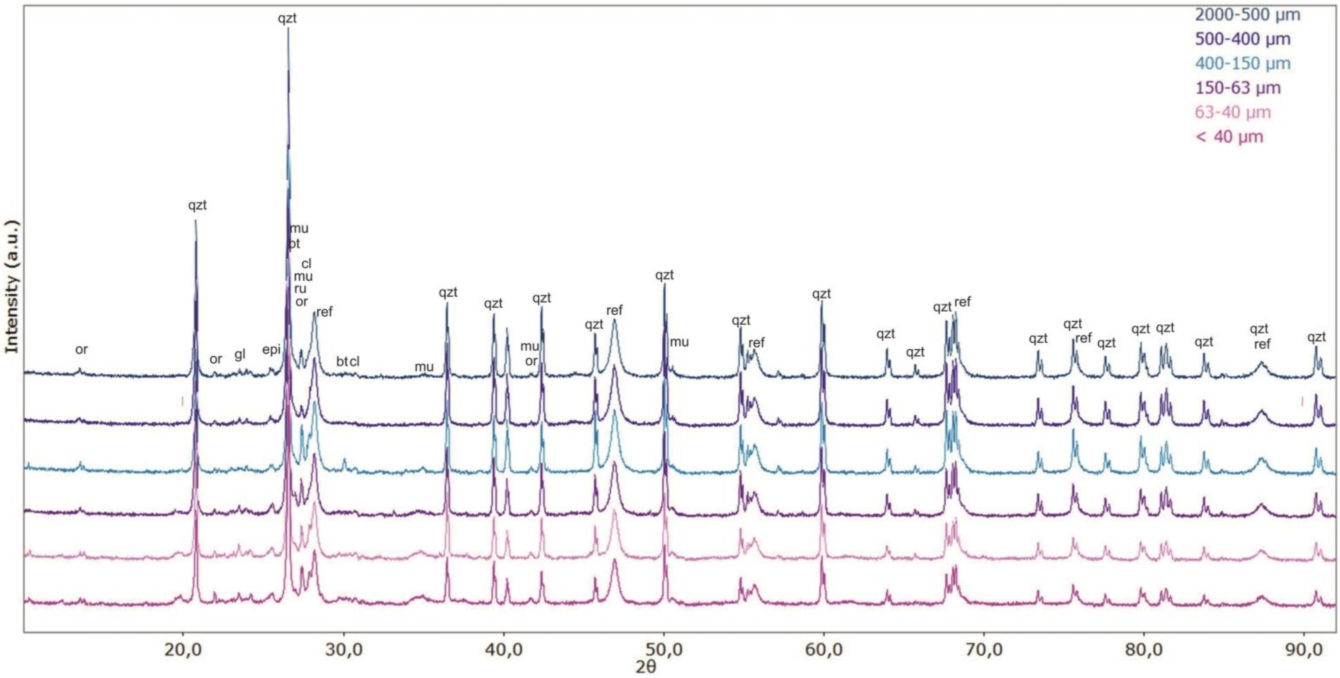

S8 Fig. Diffractograms of fractions of AR611C. Acronyms denote: bt – biotite, cl – clinochlore, epi – epidote, gl – glauconite, mu – muscovite, or – orthoclase, qzt – quartz, ref – CaF_2 and ru – rutile.

S11 Table. Properties of AR611C fractions used for the adsorption experiments.

Fraction (μm)	Mineralogical composition[a] (%)																C_{oc}[b] (%)	C_{FeOx}[c] (g/kg)	C_{AlOx}[c] (g/kg)
	Quartz	Orthoclase	Rutile	Muscovite	Titanite	Glauconite	Biotite	Kaolinite	Epidote	Illite	Albite	Clinochlore	Vermiculite	Lepidocrocite	Fluorphlogopite	Amorphous[d]			
2000-500	93.27	4.28	2.32	nd[e]	nd	nd	nd	nd	nd	nd	nd	nd	nd	nd	nd	0.13	0.03	0.52	0.35
500-400	90.95	4.97	0.94	nd	nd	nd	nd	nd	nd	nd	nd	0.17	nd	nd	nd	nd	0.01	0.27	0.25
400-150	71.59	5.34	5.70	0.85	0.90	nd	nd	nd	0.32	nd	0.28	0.11	nd	0.03	nd	14.87	0.03	0.55	0.36
150-63	78.94	7.16	5.10	1.33	0.13	0.34	nd	0.24	0.51	nd	0.24	0.31	nd	0.20	nd	5.49	0.05	1.08	0.69
63-40	59.56	11.46	4.15	3.64	0.71	0.95	0.67	0.71	0.32	0.44	0.47	0.27	0.11	0.29	nd	16.24	0.24	3.29	1.80
< 40	56.16	15.01	7.41	5.25	0.86	1.19	1.32	0.79	0.52	1.09	0.33	0.26	0.52	0.15	0.22	8.92	0.34	4.92	2.33

[a]determined using a powder X-ray diffraction technique; [b]determined using a SSM-5000A solid sample module of the Shimadzu TOC-VCSH analyser; [c]Al and Fe oxides and hydroxides determined using the Tamm's solution (for details see S2 Section and Pansu and Gautheyrou [1])); [d] amorphous phase and minerals below the detection limit; [e] amount below the detection limit.

References

1. Pansu M, Gautheyrou J. Handbook of soil analysis. Berlin, Heidelberg, New York: Springer; 2006. 993 p.
