# Supplementary material for: Adsorption of bentazone in the profiles of mineral soils with low organic matter content
Source: PLoS One. 2020 Dec 2;15(12):e0242980. doi: 10.1371/journal.pone.0242980 (PMC7710104; doi:10.1371/journal.pone.0242980)
Supplement: S9 Appendix — S12 Table. Kendall (bottom, left) and Pearson (top, right) correlation coefficients (n = 12). (PDF) [file pone.0242980.s009.pdf]

I Appendix. Adsorption in 12 selected soils with pH < 5.0 and C_{oc} < 0.35%

S12 Table. Kendall (bottom, left) and Pearson (top, right) correlation coefficients (n = 12).

	Quartz	Orthoclase	Rutile	Muscovite	Ilite	Kaolinite	Epidote	Titanite	Albite	Biotite	Glauconite	Clinochlore	Vermiculite	Lepidocrocite +Goethite	Diopside	Amorphous	C_{oc}	C_{sand}	C_{vfs+fs} 50-250	C_{Fe}	C_{Al}	K_d
Quartz		-0.539	0.268	-0.939	-0.930	-0.948	-0.081	-0.046	0.489	-0.895	-0.700	-0.180	-0.789	-0.644	0.202	-0.794	-0.322	0.949	0.644	0.013	-0.720	0.594
		0.070	0.401	<0.001	<0.001	<0.001	0.801	0.887	0.107	<0.001	0.011	0.575	0.002	0.024	0.530	0.002	0.307	<0.001	0.024	0.969	0.008	0.042
Orthoclase	-0.455		-0.163	0.604	0.438	0.572	0.600	0.482	0.193	0.670	0.590	0.530	0.503	0.438	-0.171	0.030	0.307	-0.543	0.035	-0.085	0.002	-0.234
	0.040		0.613	0.038	0.155	0.052	0.039	0.113	0.549	0.017	0.043	0.077	0.096	0.155	0.594	0.928	0.331	0.068	0.915	0.794	0.996	0.463
Rutile	0.182	-0.121		-0.457	-0.318	-0.463	-0.212	-0.145	0.550	-0.436	-0.403	-0.235	-0.366	-0.617	0.444	-0.537	-0.067	0.469	0.263	-0.087	-0.268	0.063
	0.411	0.583		0.135	0.314	0.130	0.509	0.653	0.064	0.156	0.194	0.462	0.243	0.033	0.148	0.072	0.836	0.124	0.408	0.788	0.400	0.846
Muscovite	-0.697	0.394	-0.242		0.909	0.980	0.291	0.096	-0.504	0.922	0.733	0.338	0.756	0.691	-0.166	0.739	0.254	-0.982	-0.668	-0.169	0.663	-0.550
	0.002	0.075	0.273		<0.001	<0.001	0.359	0.766	0.095	<0.001	0.007	0.282	0.005	0.013	0.606	0.006	0.425	<0.001	0.018	0.600	0.019	0.064
Illite	-0.757	0.362	-0.329	0.691		0.908	0.096	0.146	-0.518	0.864	0.677	0.187	0.861	0.536	0.029	0.754	0.085	-0.907	-0.667	-0.138	0.724	-0.672
	0.002	0.131	0.170	0.004		<0.001	0.767	0.652	0.085	<0.001	0.016	0.560	<0.001	0.072	0.928	0.005	0.793	<0.001	0.018	0.669	0.008	0.017
Kaolinite	-0.509	0.381	-0.477	0.699	0.500		0.169	0.173	-0.539	0.912	0.706	0.200	0.792	0.723	-0.269	0.774	0.253	-0.995	-0.631	-0.111	0.697	-0.567
	0.028	0.100	0.040	0.003	0.047		0.601	0.591	0.071	<0.001	0.010	0.533	0.002	0.008	0.399	0.003	0.428	<0.001	0.028	0.731	0.012	0.055
Epidote	-0.121	0.424	-0.030	0.242	0.329	0.064		0.010	0.162	0.226	0.232	0.791	0.001	0.298	0.359	-0.213	0.361	-0.186	-0.039	-0.185	-0.280	-0.192
	0.583	0.055	0.891	0.273	0.170	0.784		0.975	0.616	0.480	0.469	0.002	0.998	0.346	0.252	0.506	0.248	0.562	0.905	0.564	0.377	0.550
Titanite	0.055	0.385	-0.239	-0.018	-0.040	0.193	0.018		0.338	0.176	0.118	-0.099	0.476	-0.068	-0.190	-0.230	-0.401	-0.091	0.369	0.018	-0.135	0.077
	0.837	0.150	0.373	0.945	0.891	0.493	0.945		0.283	0.585	0.715	0.759	0.118	0.834	0.554	0.472	0.196	0.779	0.238	0.956	0.675	0.813
Albite	0.364	0.061	0.394	-0.424	-0.329	-0.477	0.212	0.275		-0.495	-0.432	0.105	-0.448	-0.511	0.238	-0.795	-0.144	0.584	0.736	0.156	-0.463	0.473
	0.100	0.784	0.075	0.055	0.170	0.040	0.337	0.304		0.102	0.161	0.745	0.145	0.090	0.457	0.002	0.656	0.046	0.006	0.629	0.130	0.121
Biotide	-0.716	0.532	-0.422	0.679	0.737	0.674	0.239	0.133	-0.385		0.900	0.283	0.815	0.608	-0.264	0.656	0.235	-0.921	-0.535	-0.273	0.501	-0.576
	0.008	0.047	0.115	0.011	0.011	0.016	0.373	0.681	0.150		<0.001	0.373	0.001	0.036	0.407	0.021	0.462	<0.001	0.073	0.391	0.097	0.050

Glauconite	-0.569	0.459	-0.422	0.532	0.578	0.597	0.239	0.044	-0.385	0.822		0.307	0.691	0.411	-0.234	0.492	0.314	-0.731	-0.360	-0.396	0.241	-0.400
	**0.034**	0.087	0.115	**0.047**	**0.047**	**0.034**	0.373	0.891	0.150	**0.011**		0.333	**0.013**	0.184	0.464	0.104	0.321	**0.007**	0.250	0.202	0.451	0.198
Clinochlore	-0.107	0.412	0.199	0.260	0.199	0.032	0.657	-0.222	0.199	0.129	0.203		0.001	0.398	0.316	-0.049	0.231	-0.205	0.058	-0.159	-0.029	0.071
	0.631	0.064	0.373	0.244	0.411	0.891	**0.003**	0.411	0.373	0.631	0.451		0.998	0.200	0.317	0.881	0.471	0.523	0.858	0.623	0.930	0.826
Vermiculite	-0.584	0.270	-0.450	0.539	0.683	0.566	-0.045	0.327	-0.405	0.762	0.599	-0.181		0.355	-0.179	0.594	-0.035	-0.768	-0.496	-0.057	0.412	-0.530
	0.075	0.411	0.170	0.100	0.055	0.100	0.891	0.411	0.217	0.055	0.131	0.583		0.257	0.577	**0.042**	0.915	**0.004**	0.101	0.860	0.184	0.077
Lepidocrocite +Goethite	-0.509	0.381	-0.413	0.477	0.500	0.617	0.318	0.000	-0.350	0.520	0.366	0.096	0.377		-0.375	0.667	0.490	-0.729	-0.308	0.202	0.562	-0.369
	**0.028**	0.100	0.075	**0.040**	**0.047**	**0.011**	0.170	1.000	0.131	0.064	0.193	0.681	0.273		0.229	**0.018**	0.106	**0.007**	0.331	0.530	0.057	0.239
Diopside	0.349	-0.188	-0.027	-0.188	-0.029	-0.282	0.242	0.163	0.349	-0.325	-0.325	0.135	-0.239	-0.225		-0.357	-0.205	0.251	-0.055	-0.323	-0.131	-0.215
	0.373	0.631	0.945	0.631	0.945	0.493	0.537	0.732	0.373	0.493	0.493	0.732	0.681	0.583		0.254	0.523	0.431	0.865	0.306	0.685	0.503
Amorphous	-0.576	0.152	-0.485	0.455	0.494	0.509	-0.061	-0.128	-0.485	0.532	0.459	-0.168	0.584	0.477	-0.510		0.262	-0.796	-0.731	0.173	0.817	-0.448
	**0.009**	0.493	**0.028**	**0.040**	**0.040**	**0.028**	0.784	0.631	**0.028**	**0.047**	0.087	0.451	0.075	**0.040**	0.193		0.411	**0.002**	**0.007**	0.591	**0.001**	0.144
C_{oc}	-0.424	0.303	-0.030	0.182	0.263	0.159	0.212	-0.239	-0.091	0.312	0.459	0.199	0.090	0.318	-0.296	0.303		-0.298	-0.121	0.284	0.005	-0.227
	0.055	0.170	0.891	0.411	0.273	0.493	0.337	0.373	0.681	0.244	0.087	0.373	0.784	0.170	0.451	0.170		0.347	0.708	0.371	0.988	0.478
C_{sand}	0.576	-0.152	0.424	-0.758	-0.658	-0.667	-0.182	0.128	0.606	-0.642	-0.569	-0.015	-0.539	-0.509	0.188	-0.576	-0.364		0.675	0.141	-0.695	0.609
	**0.009**	0.493	0.055	**0.001**	**0.006**	**0.004**	0.411	0.631	**0.006**	**0.016**	**0.034**	0.945	0.100	**0.028**	0.631	**0.009**	0.100		**0.016**	0.662	**0.012**	**0.036**
Cvfs+fs 50-250	0.455	0.030	0.182	-0.576	-0.592	-0.350	0.000	0.275	0.546	-0.422	-0.275	0.107	-0.450	-0.254	0.188	-0.455	-0.121	0.697		0.174	-0.542	0.627
	**0.040**	0.891	0.411	**0.009**	**0.014**	0.131	1.000	0.304	**0.014**	0.115	0.304	0.631	0.170	0.273	0.631	**0.040**	0.583	**0.002**		0.588	0.068	**0.029**
C_{Fe}	-0.030	-0.091	0.000	-0.152	-0.132	-0.095	-0.303	0.165	0.061	-0.202	-0.275	-0.321	0.090	0.032	-0.296	0.212	-0.061	0.152	0.030		0.102	0.200
	0.891	0.681	1.000	0.493	0.583	0.681	0.170	0.537	0.784	0.451	0.304	0.150	0.784	0.891	0.451	0.337	0.784	0.493	0.891		0.752	0.534
C_{Al}	-0.697	0.333	-0.242	0.879	0.658	0.636	0.121	-0.018	-0.364	0.679	0.532	0.137	0.584	0.381	-0.296	0.515	0.121	-0.636	-0.515	-0.091		-0.378
	**0.002**	0.131	0.273	**<0.001**	**0.006**	**0.006**	0.583	0.945	0.100	**0.011**	**0.047**	0.537	0.075	0.100	0.451	**0.020**	0.583	**0.004**	**0.020**	0.681		0.226
K_d	0.424	-0.121	0.212	-0.424	-0.658	-0.254	-0.152	-0.092	0.212	-0.532	-0.385	0.076	-0.629	-0.318	-0.027	-0.303	-0.212	0.546	0.546	0.061	-0.364	
	0.055	0.583	0.337	0.055	**0.006**	0.273	0.493	0.732	0.337	**0.047**	0.150	0.732	0.055	0.170	0.945	0.170	0.337	**0.014**	**0.014**	0.784	0.100	
