# Supplementary material for: Adsorption of bentazone in the profiles of mineral soils with low organic matter content
Source: PLoS One. 2020 Dec 2;15(12):e0242980. doi: 10.1371/journal.pone.0242980 (PMC7710104; doi:10.1371/journal.pone.0242980)
Supplement: S10 Appendix — S9 Fig. Molecular electrostatic potentials (MEPs) of bentazone. The deepest blue color denotes the most positive potential, the deepest red color–the most negative potential, and intermediate shades–the intermediate potential regions (calculated at RHF 6–311+G** basis set). S10 Fig. The charge distribution of the ketone (a, b) and enol (c, d) tautomers of bentazone: Mullicen charge (a, c), electrostatic charge (b, d). Calculations at RHF 6–311+G** basis set. S13 Table. Total energy (ET (au)) of bentazone tautomers calculated on different ways. (PDF) [file pone.0242980.s010.pdf]

J Appendix. Modeling of bentazone adsorption on quartz surface

Table S13. Total energy (E_T (au)) of bentazone tautomers calculated on different ways.

Form	PM3	RHF 6-311+G**	RHF STO-3G (water)	DFT/B3LYP/ 6-311++G(d,p)
Ketone form	-0.1433	-1116.81	-1102.95	-1121.93
Enol form	-0.1118	-1116.76	-1102.89	-1116.75

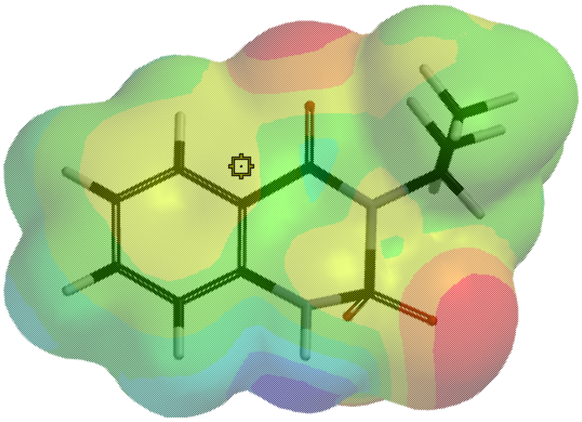

Fig. S9. Molecular electrostatic potentials (MEPs) of bentazone. The deepest blue color denotes the most positive potential, the deepest red color–the most negative potential, and intermediate shades–the intermediate potential regions (calculated at RHF 6-311+G** basis set).

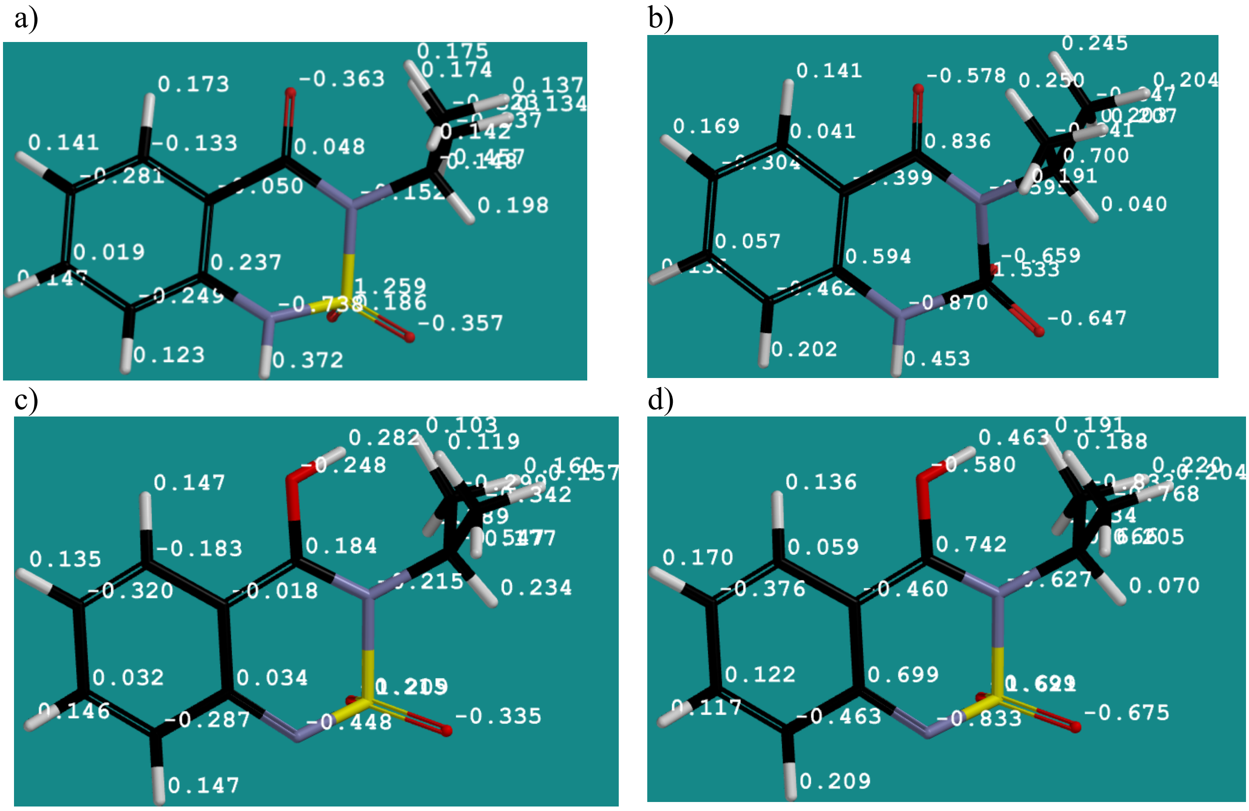

Fig. S10. The charge distribution of the ketone (a, b) and enol (c, d) tautomers of bentazone: Mullicen charge (a, c), electrostatic charge (b, d). Calculations at RHF 6-311+G** basis set.
